# *Dhr96[1]* mutation and maternal *tudor[1]* mutation increase life span and reduce the beneficial effects of mifepristone in mated female Drosophila

**DOI:** 10.1371/journal.pone.0292820

**Published:** 2023-12-21

**Authors:** Gary N. Landis, Hans S. Bell, Oscar Peng, Brett Bognar, Andy Tong, Tomás D. Manea, Hanmei Bao, Xianlin Han, John Tower

**Affiliations:** 1 Department of Biological Sciences, University of Southern California, Los Angeles, California, United States of America; 2 Barshop Institute for Longevity and Aging Studies, University of Texas Health Science Center at San Antonio, San Antonio, Texas, United States of America; 3 Department of Medicine, University of Texas Health Science Center at San Antonio, San Antonio, Texas, United States of America; University of Mississippi, UNITED STATES

## Abstract

Mating and receipt of male Sex Peptide hormone cause increased egg laying, increased midgut size and decreased life span in female Drosophila. Feeding mated females with the synthetic steroid mifepristone decreases egg production, reduces midgut size, and increases life span. Here, several gene mutations were assayed to investigate possible mechanisms for mifepristone action. Drosophila Dhr96 is a hormone receptor, and a key positive regulator of midgut lipid uptake and metabolism. *Dhr96[1]* null mutation increased female life span, and reduced the effects of mifepristone on life span, suggesting that *Dhr96[1]* mutation and mifepristone may act in part through the same mechanism. Consistent with this idea, lipidomics analysis revealed that mating increases whole-body levels of triglycerides and fatty-acids in triglycerides, and these changes are reversed by mifepristone. Maternal *tudor[1]* mutation results in females that lack the germ-line and produce no eggs. Maternal *tudor[1]* mutation increased mated female life span, and reduced but did not eliminate the effects of mating and mifepristone on life span. This indicates that decreased egg production may be related to the life span benefits of mifepristone, but is not essential. Mifepristone increases life span in *w[1118]* mutant mated females, but did not increase life span in *w[1118]* mutant virgin females. Mifepristone decreased egg production in *w[1118]* mutant virgin females, indicating that decreased egg production is not sufficient for mifepristone to increase life span. Mifepristone increases life span in virgin females of some, but not all, *white[+]* and *mini-white[+]* strains. Backcrossing of *mini-white[+]* transgenes into the *w[1118]* background was not sufficient to confer a life span response to mifepristone in virgin females. Taken together, the data support the hypothesis that mechanisms for mifepristone life span increase involve reduced lipid uptake and/or metabolism, and suggest that mifepristone may increase life span in mated females and virgin females through partly different mechanisms.

## Introduction

Mifepristone (RU486) is a synthetic steroid with a long history of safe and effective use in humans [1, 2]. Mifepristone antagonizes the progesterone receptor, enabling its use in birth control. In addition, mifepristone antagonizes the activity of the type II glucocorticoid receptor, enabling its use as treatment for Cushing’s disease [1, 2]. Mifepristone is also reported to be a mammalian PPARγ agonist that activates expression of PPARγ target genes [3, 4]. Several studies report anti-obesity and anti-diabetic effects of mifepristone in humans and mice [5–8]. Mifepristone reduced fasting-stimulated lipolysis in mice [9], and reduced glucocorticoid-stimulated lipolysis in cultured rat adipocytes [10]. Consistent with this, short-term treatment with 5mg/kg mifepristone reduced serum triglyceride levels and improved adipose and hepatic insulin sensitivity in obese human patients with hyperglycemia [7]. Both the glucocorticoid receptor and PPARγ are implicated as potential targets for mifepristone’s anti-obesity and anti-diabetes effects in mammals, and it is possible that additional relevant mifepristone targets remain to be identified.

Mifepristone also exhibits striking physiological effects in Drosophila. In female Drosophila, mating and male Sex Peptide (SP) hormone cause increased juvenile hormone (JH) and ecdysone hormone levels. JH and ecdysone induce midgut hypertrophy, inflammation markers, and increased amino acid (AA) and lipid metabolism [11–13], which supports increased egg production [14–17]. The midgut hypertrophy, increased AA metabolites and lipids, inflammation, and decreased survival caused by mating and SP can each be reversed by feeding mated females mifepristone, yielding +100% increase in median life span in long-lived strains [18–20]. Mifepristone also increased life span of virgin females by +16–30%, on both normal and high-fat diet (HFD), in long-lived strains [21]. In both mated and virgin females, mifepristone decreased whole-body levels of several lipids and numerous AA metabolites [21]. The effect of mifepristone on food intake has been assayed using the dye-uptake assay [19], the CAFÉ assay [18], and the excrement quantification (EXQ) assay [20, 21]. Remarkably, mifepristone did not reduce food intake in either mated or virgin females, on normal or HFD, and was often associated with increased food intake, suggesting a possible compensatory response to reduced nutrient absorption or metabolism. In contrast, under extremely low nutrient conditions, mifepristone has been reported to have an aversive effect, which may be relevant to studies of dietary restriction [22]. Effects of mifepristone in Drosophila do not require presence of the Gene-Switch transcription factor [18, 19]. In addition, mifepristone has no detectable antibiotic activity in vivo or in vitro, and mifepristone increases life span of mated females under both normal and axenic conditions [23, 24]. The Drosophila midgut has been well-characterized in terms of its anatomy and function in uptake of nutrients, including lipids [25–28]. Because of its molecular and genetic tractability, Drosophila provides an ideal model in which to investigate the mechanisms of mifepristone, including identifying potential mifepristone receptors. Drosophila contains orthologs for all major subclasses of vertebrate receptors [29].

Mifepristone reduces progeny production in mated Drosophila females [19], suggesting the possibility that some or all of the life span benefits of mifepristone might be due to reduced generation of eggs. To begin to address this possibility, flies were analyzed where the germ-line cells were ablated using a maternal *tudor[1]* mutation [30, 31]. Tudor is required for specification of germ-line cells in developing eggs, and in its absence the eggs develop into sterile adults that lack the germ-line. Here these sterile females were found to still exhibit modestly increased life span in response to mifepristone, indicating that reduced egg production is not strictly required for mifepristone-induced life span extension. The Drosophila *Dhr96* gene encodes a hormone receptor that functions in the midgut to regulate lipid uptake and metabolism [32–34]. Dhr96 protein is orthologous to several human hormone receptors, including the Vitamin D receptor and the LXR (NR1H) receptor [29, 35]. *Dhr96[1]* mutant phenotypes include reduced whole-body triglyceride levels, and a modestly increased sensitivity to xenobiotics such as DDT. Dhr96 protein is reported to bind cholesterol as a ligand [35], and given the similarity in structure between cholesterol and mifepristone, we hypothesized that Dhr96 might be a mifepristone target. Here, the *Dhr96[1]* mutation is found to increase life span, and to reduce the effects of mating and mifepristone on life span in female Drosophila, suggesting that mifepristone and *Dhr96[1]* may act in part through the same mechanism. Consistent with this possibility, lipidomics analysis reveals that mifepristone reduces whole-body levels of triglycerides and fatty-acids. Taken together, the data implicate reduced midgut lipid uptake and/or metabolism as one likely mechanism for mifepristone life span extension.

## Results

### Effect of *Dhr96[1]* mutation on life span and the response to mating and mifepristone

The *Dhr96[1]* null mutation was generated using the “ends-in” targeting method, which yielded a deletion of *Dhr96* exon 4, and the insertion of the *3xP3-eGFP* marker gene that produces expression of eGFP in the eye [32, 36]. The *Dhr96[1]* mutation was backcrossed for 9 generations into the *w[1118]* reference strain background, and made homozygous by scoring the *3xP3-eGFP* marker (S1A–S1C Fig). The presence of the expected 331bp deletion of exon 4 was confirmed by PCR (S1D–S1F Fig). Consistent with previous reports [32, 37], the *Dhr96[1]* strain showed modestly increased sensitivity to DDT toxicity relative to the *w[1118]* control strain (S1G Fig).

**Fig 1:**
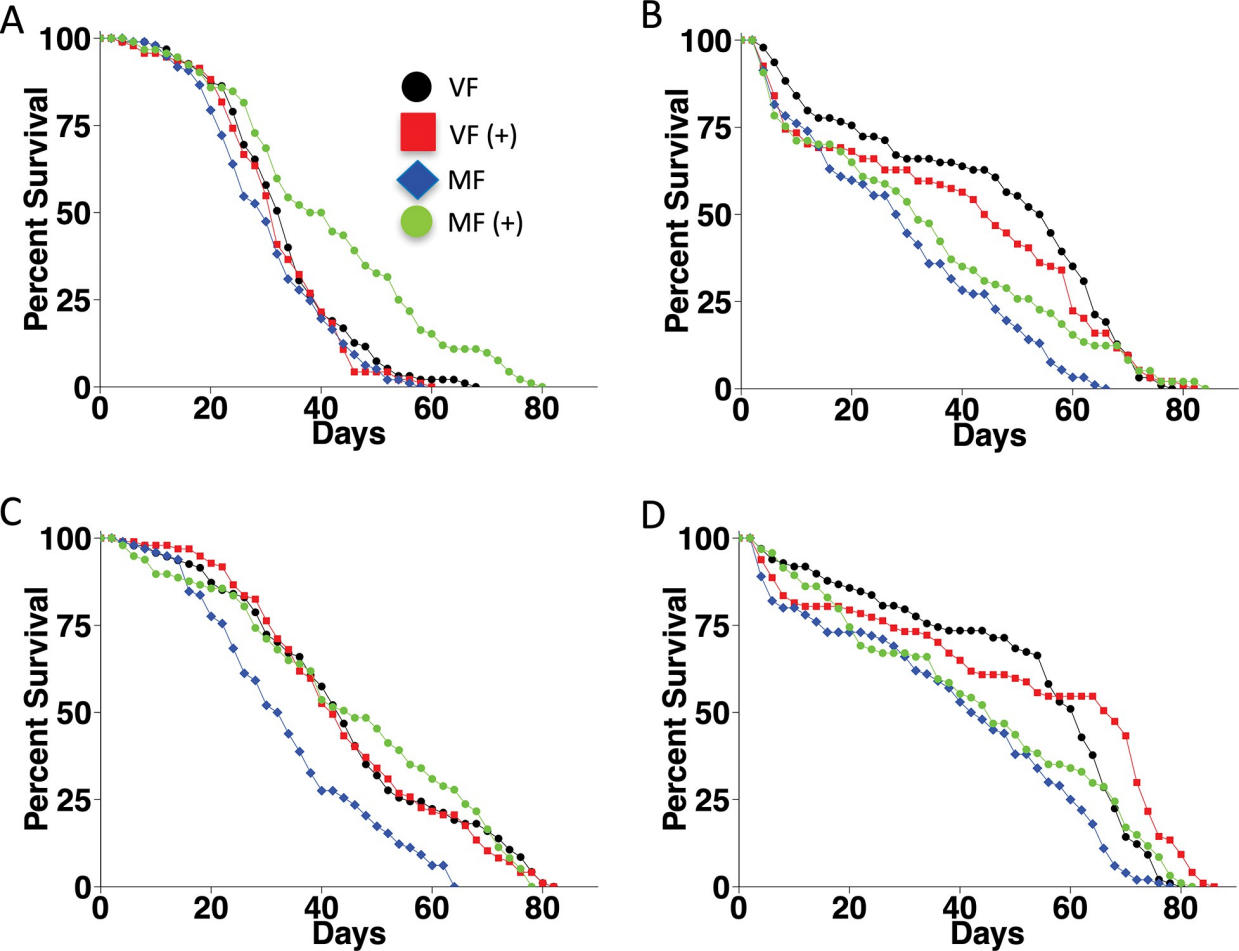
*Dhr96[1]* mutation effect on life span and response to mifepristone. A. *w[1118]* strain, experiment 1. B*. Dhr96[1]* strain, experiment 1. C. *w[1118]* strain, experiment 2. D. *Dhr96[1]* strain, experiment 2. VF, virgin female. MF, mated female. (+), 200μg/ml mifepristone. Statistical summaries presented in Table 1.

**Fig 2:**
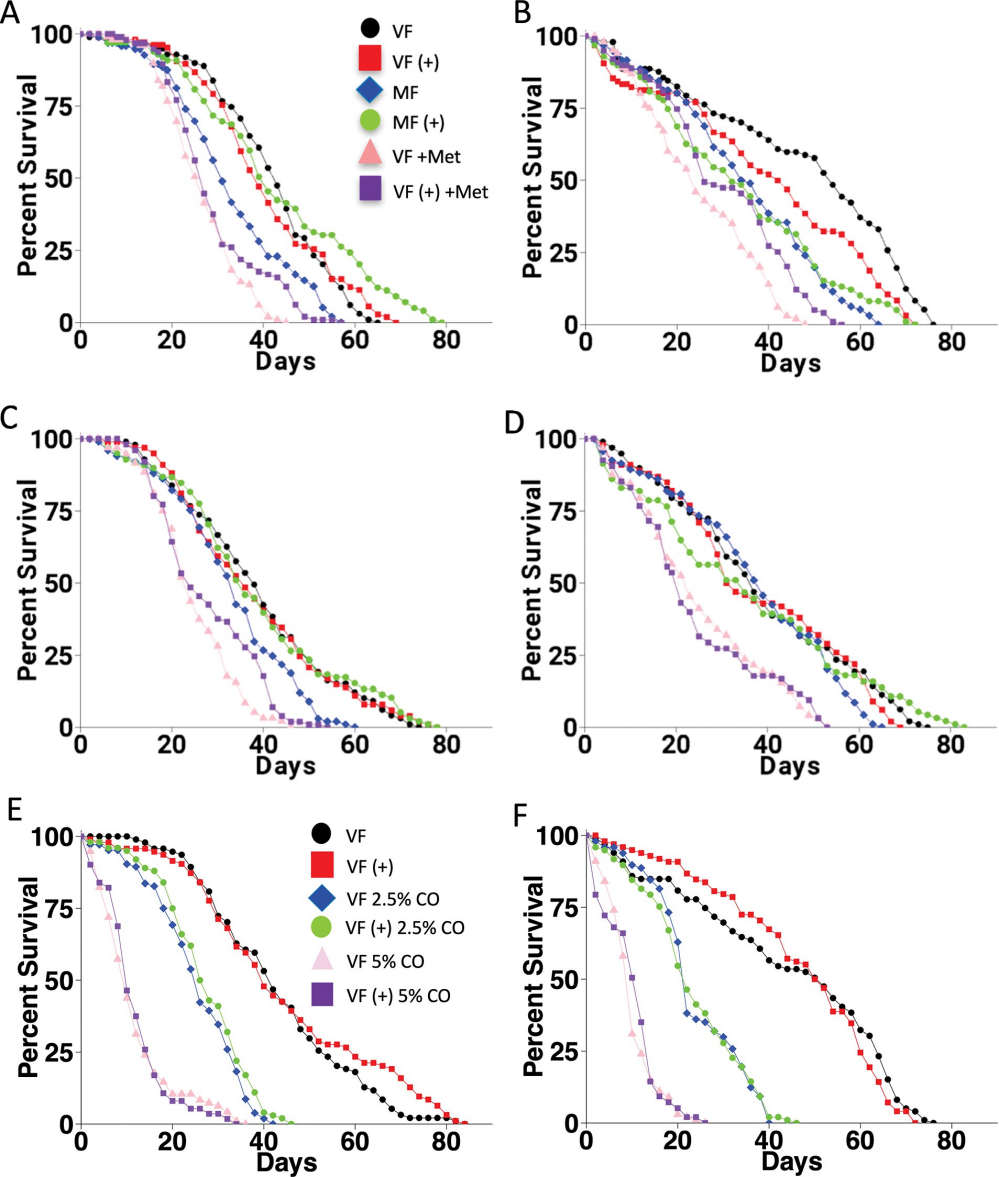
*Dhr96[1]* mutation effect on life span and response to mifepristone, methoprene and HFD. (A-D) Life span assay and effect of methoprene. VF, virgin female. MF, mated female. (+), 200μg/ml mifepristone. +Met, 100μg/ml methoprene. A. *w[1118]* strain, experiment 1. B. *Dhr96[1]* strain, experiment 1. C. *w[1118]* strain, experiment 2. D. *Dhr96[1]* strain, experiment 2. Statistical summaries presented in Table 1. (E, F) Life span assay and effect of HFD. Virgin female (VF) life span was assayed in absence and presence of the indicated concentrations of coconut oil (CO). E. *w[1118]* control strain. F. *Dhr96[1]* strain. Statistical summaries presented in Table 2.

The *Dhr96[1]* strain and the *w[1118]* control strain were assayed for life span in virgin females and mated females, in the presence and absence of mifepristone, in four replicate experiments (Fig 1A–1D; Fig 2A–2D; Table 1). Consistent with previous assays of the *w[1118]* strain [19], COX proportional hazard analysis (COX-PHA) showed a significant effect of mating and a significant interaction between mating and mifepristone (S1 Table). Mating decreased life span, with decreases ranging from -11% to -30%, and mifepristone increased life span in the mated females, with increases ranging from +6% to +40% (Table 1). In addition, COX-PHA revealed a significant effect of *Dhr96[1]* mutation on life span (S1 Table). Consistent with that result, pair-wise comparisons show that *Dhr96[1]* mutation significantly increased life span in virgin females in 2/4 assays, and significantly increased life span in mated females in 3/4 assays, and no significant decreases due to *Dhr96[1]* mutation were observed (Table 1). Finally, COX-PHA also revealed a significant interaction between *Dhr96[1]* mutation and mating (S1 Table). *Dhr96[1]* mutation reduced the beneficial effect of mifepristone in mated females relative to its effects in the *w[1118]* controls: Mifepristone significantly increased life span in *w[1118]* mated females in 4/4 assays, with increases ranging from +6% to +39%. In contrast, mifepristone significantly increased life span in *Dhr96[1]* mated females in only 1/4 assays, with an increase of +7% (Table 1).

**Table 1:** Effect of *Dhr96[1]* mutation on life span in response to mating, mifepristone and methoprene.

Experiment	Genotype	Sex/status	Drug	N	Med	90% Mort	ΔMed^a^ (%)	*p* ^a^	ΔMed^b^ (%)	*p* ^b^
1	*w[1118]*	VF	-	95	34	50				
1	*w[1118]*	VF	(+)	93	32	46	-5.88	0.3857		
1	*w[1118]*	MF	-	97	30	46	-11.8	0.1258		
1	*w[1118]*	MF	(+)	92	40	69	33.3	6.16E-8		
1	*Dhr96[1]*	VF	-	94	55	70			61.8	8.93E-10
1	*Dhr96[1]*	VF	(+)	94	45	70	-18.2	0.3597		
1	*Dhr96[1]*	MF	-	92	29	56	-47.3	1.53E-9	-3.33	0.1812
1	*Dhr96[1]*	MF	(+)	97	32	70	10.3	0.0194		
2	*w[1118]*	VF	-	94	44	75				
2	*w[1118]*	VF	(+)	97	42	71	-4.54	0.7462		
2	*w[1118]*	MF	-	98	33	58	-25.0	2.55e-5		
2	*w[1118]*	MF	(+)	97	46	74	39.4	1.60E-7		
2	*Dhr96[1]*	VF	-	98	62	74			40.9	0.1061
2	*Dhr96[1]*	VF	(+)	97	68	80	9.68	0.0029		
2	*Dhr96[1]*	MF	-	100	43	68	-30.6	7.58E-5	30.3	0.0001
2	*Dhr96[1]*	MF	(+)	94	46	76	6.98	0.0106		
3	*w[1118]*	VF	-	97	43	59				
3	*w[1118]*	VF	(+)	96	39	63	-9.30	0.7888		
3	*w[1118]*	MF	-	96	31	53	-27.9	4.78E-6		
3	*w[1118]*	MF	(+)	99	41	69	32.3	5.39E-7		
3	*w[1118]*	VF	Met	100	27	39	-37.2	1.76E-21		
3	*w[1118]*	VF	Met (+)	99	27	47	0.00	0.0253		
3	*Dhr96[1]*	VF	-	97	54	72			25.5	1.0E-6
3	*Dhr96[1]*	VF	(+)	96	43	67	-20.4	0.0018		
3	*Dhr96[1]*	MF	-	96	35	56	-35.2	8.22E-10	71.0	1.10E-11
3	*Dhr96[1]*	MF	(+)	99	32	60	-8.57	0.5734		
3	*Dhr96[1]*	VF	Met	100	24	42	-55.6	4.80E-18		
3	*Dhr96[1]*	VF	Met (+)	99	26	48	8.33	0.0004		
4	*w[1118]*	VF	-	99	38	62				
4	*w[1118]*	VF	(+)	101	36	62	-5.26	0.9344		
4	*w[1118]*	MF	-	101	34	50	-10.5	0.0012		
4	*w[1118]*	MF	(+)	98	36	69	5.88	0.0031		
4	*w[1118]*	VF	Met	96	24	36	-36.8	1.81E-13		
4	*w[1118]*	VF	Met (+)	101	24	42	0.00	0.0189		
4	*Dhr96[1]*	VF	-	98	37	67			0.00	0.3261
4	*Dhr96[1]*	VF	(+)	100	32	63	-13.5	0.3141		
4	*Dhr96[1]*	MF	-	94	39	59	5.41	0.0920	14.7	0.0001
4	*Dhr96[1]*	MF	(+)	94	35	70	-10.3	0.3353		
4	*Dhr96[1]*	VF	Met	97	23	47	-37.8	1.41E-8		
4	*Dhr96[1]*	VF	Met (+)	95	21	49	-8.70	0.9583		

(+), 200μg/ml mifepristone. Met, 100μg/ml methoprene. (a) In each experiment, MF (-) is compared to VF (-) to determine effect of mating, and for each sex/status, (+) is compared to (-) to determine effect of mifepristone. In experiments 3 and 4, Met is compared to (-) to determine effect of Met, and Met (+) is compared to Met to determine effect of mifepristone in the presence of Met. (b) In each experiment, VF(-) *w[1118]* is compared to VF(-) *Dhr96[1]*, and MF(-) *w[1118]* is compared to MF(-) *Dhr96[1]*, to determine the life span effect of *Dhr96[1]* mutation. Med, median. Statistical test is log-rank, and the *p* value for significance with three comparisons is *p* ≤ 0.0167.

### Effect of methoprene and HFD on life span in *Dhr96[1]* and *w[1118]* strains

Methoprene is a juvenile hormone analog that has previously been shown to reduce life span in virgin females [38], and this negative effect was partially rescued by mifepristone in a long-lived genotype (progeny of *w[1118]* x *yw;ElavGS*) [20]. Here methoprene was assayed for effect on virgin female life span in the *Dhr96[1]* strain and the *w[1118]* control strain, in presence and absence of mifepristone, in two replicate experiments (Fig 2A–2D; Table 1). Consistent with previous studies, methoprene reduced virgin female life span, with decreases ranging from -37% to -56%; however, no rescue by mifepristone was observed in *w[1118]* strain, and no consistent rescue was observed in the *Dhr96[1]* strain (Table 1).

A HFD, containing either 2.5% or 5% coconut oil (CO), was assayed for effect on virgin female life span in the *Dhr96[1]* and *w[1118]* control strains, in the presence and absence of mifepristone (Fig 2E and 2F). COX-PA revealed a significant effect of CO, and a significant interaction between CO and *Dhr96[1]* mutation (S2 Table). CO decreased life span in a dose-dependent manner, and had slightly greater effect in *Dhr96[1]* strain relative to the *w[1118]* control strain (Table 2). Notably, no rescue of the decreased life span caused by HFD was observed for mifepristone in either genotype (Fig 2E and 2F; Table 2).

**Table 2:** Effect of *Dhr96[1*] mutation on life span in response to HFD and mifepristone.

Genotype	Sex/status	Drug/diet	N	Med	ΔMed (%)	*p*
*w[1118]*	VF	-	94	42		
*w[1118]*	VF	(+)	94	40	-4.76	0.2515
*w[1118]*	VF	2.5% CO	104	26	-38.1	6.19E-19
*w[1118]*	VF	2.5% CO (+)	100	27	3.85	0.1129
*w[1118]*	VF	5% CO	96	10	-79.2	7.48E-43
*w[1118]*	VF	5% CO (+)	112	10	0.00	0.9749
*Dhr96[1]*	VF	-	99	52		
*Dhr96[1]*	VF	(+)	98	51	-1.92	0.3681
*Dhr96[1]*	VF	2.5% CO	97	22	-57.7	5.19E-16
*Dhr96[1]*	VF	2.5% CO (+)	97	22	0.00	0.9005
*Dhr96[1]*	VF	5% CO	100	10	-83.3	1.28E-45
*Dhr96[1]*	VF	5% CO (+)	97	12	20.0	0.4131

(+), 200μg/ml mifepristone. CO, coconut oil. VF of *Dhr96[1]* are compared to VF of *w[1118]* to show effect of *Dhr96[1]* mutation. For each genotype and diet, CO is compared to (-) to determine effect of CO, and (+) is compared to (-) to determine effect of mifepristone. The statistical test is log-rank, and the p value for significance with 4 comparisons is p ≤ 0.0125.

### *Dhr96[1]* mutation effect on egg laying

**Fig 3:**
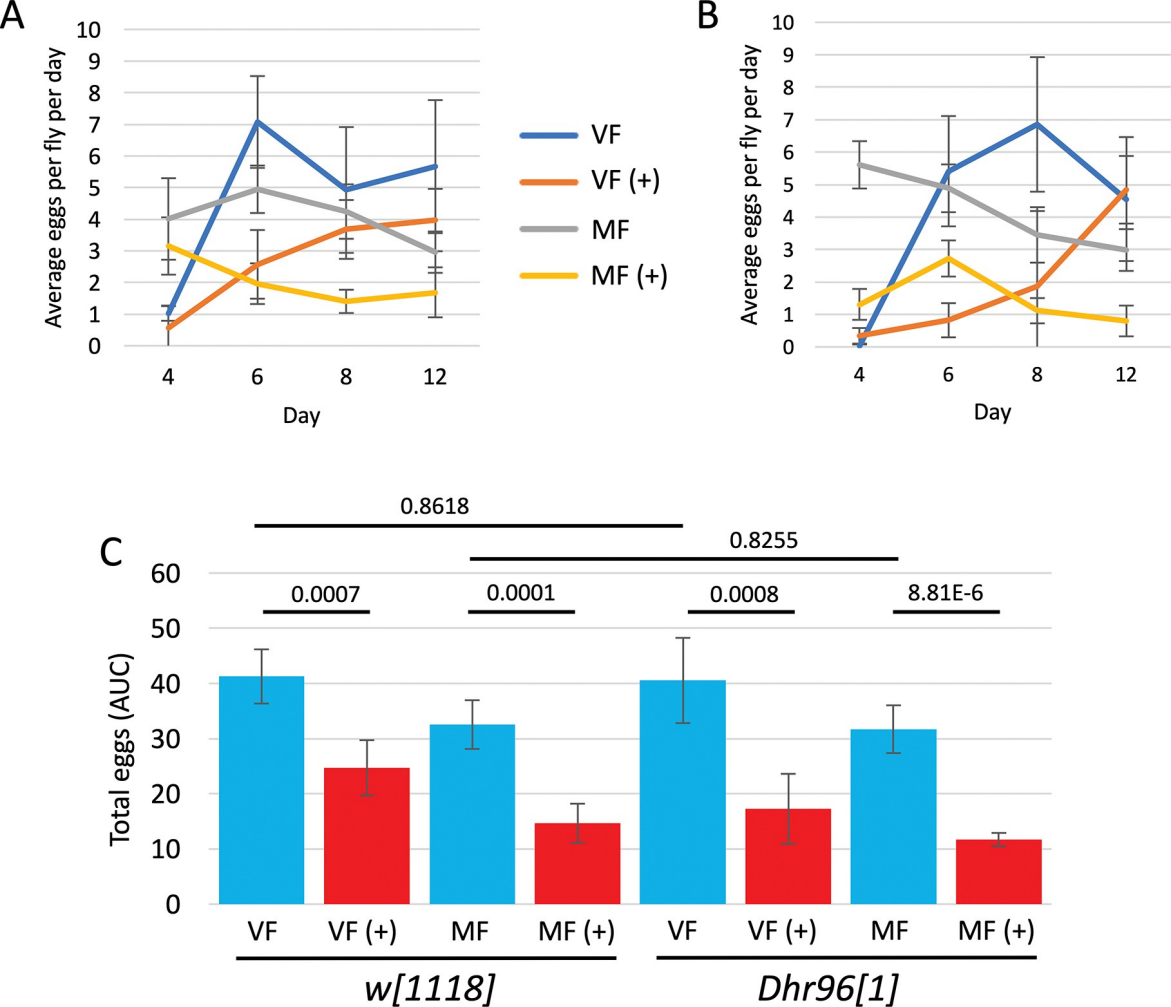
*Dhr96[1]* mutation effect on egg laying. (A, B) Average eggs per fly per day. A. *w[1118]* strain. B. *Dhr96[1]* strain. C. Total eggs per fly, estimated by area-under-curve. The statistical test is unpaired, two-sided t test, and the *p* value for significance with two comparisons is *p* ≤ 0.025. The *p* value for pair-wise comparisons is presented above the plots. VF, virgin female. MF, mated female. (+), 200μg/ml mifepristone.

Egg laying was assayed in virgin females and mated females of the *Dhr96[1]* strain and *w[1118]* control strain, in the presence and absence of mifepristone, at days 4, 6, 8 and 12 of drug treatment (Fig 3A and 3B), and total eggs laid was estimated using area-under-curve (Fig 3C). Mifepristone decreased egg production in both virgin females and mated females, in both genotypes, and no significant effect of the *Dhr96[1]* mutation was detected.

### *Dhr96[1]* mutation effect on midgut diameter

**Fig 4:**
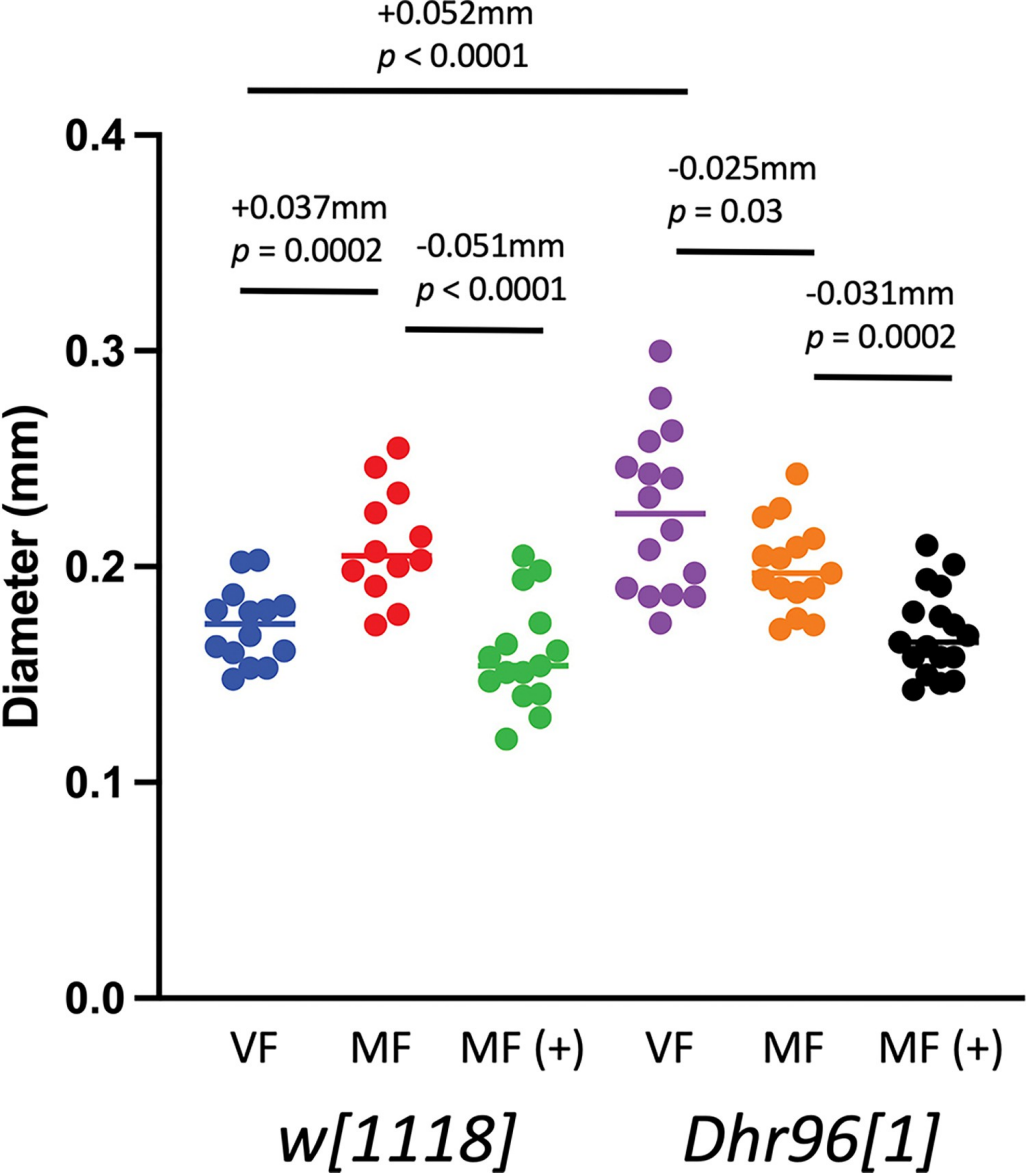
*Dhr96[1]* mutation effect on maximum midgut diameter. Maximum midgut diameter was assayed in virgin females, mated females, and mated females treated with mifepristone for both the *w[1118]* control strain and the *Dhr96[1]* strain. The statistical test is unpaired, two-sided t test, and the *p* value for significance with three comparisons is *p* ≤ 0.0167. The change in mean maximum midgut diameter between samples and the *p* value for significance is presented above the plots. VF, virgin female. MF, mated female. (+), 200μg/ml mifepristone.

Maximum midgut diameter was assayed in virgin females, mated females, and mated females treated with mifepristone (Fig 4). Midgut diameter was assayed in flies at 14 days of age, which corresponds to 12 days of treatment +/- mifepristone. This time point was selected because this is the age where life span curves of mated females treated +/- mifepristone typically begin to diverge, and this time point has previously been found to show robust mating-induced midgut hypertrophy that is reversed by mifepristone treatment [21, 39]. In the *w[1118]* control strain, mating caused increased midgut diameter, and this was significantly decreased by mifepristone treatment, consistent with previous assays of the *w[1118]* strain and other strains [21]. A surprisingly different pattern was observed in the *Dhr96[1]* strain. Increased and more variable midgut diameter was observed in virgin females of the *Dhr96[1]* strain relative to the *w[1118]* strain, and mating was associated with a small decrease instead of an increase; however, this decrease was not significant after control for multiple comparisons (Fig 4). In contrast, the effect of mifepristone was still observed in the *Dhr96[1]* strain, where it decreased midgut diameter in mated females, similar to the effect of mifepristone observed in the *w[1118]* strain.

### Effect of mifepristone on whole-body lipid levels

**Fig 5:**
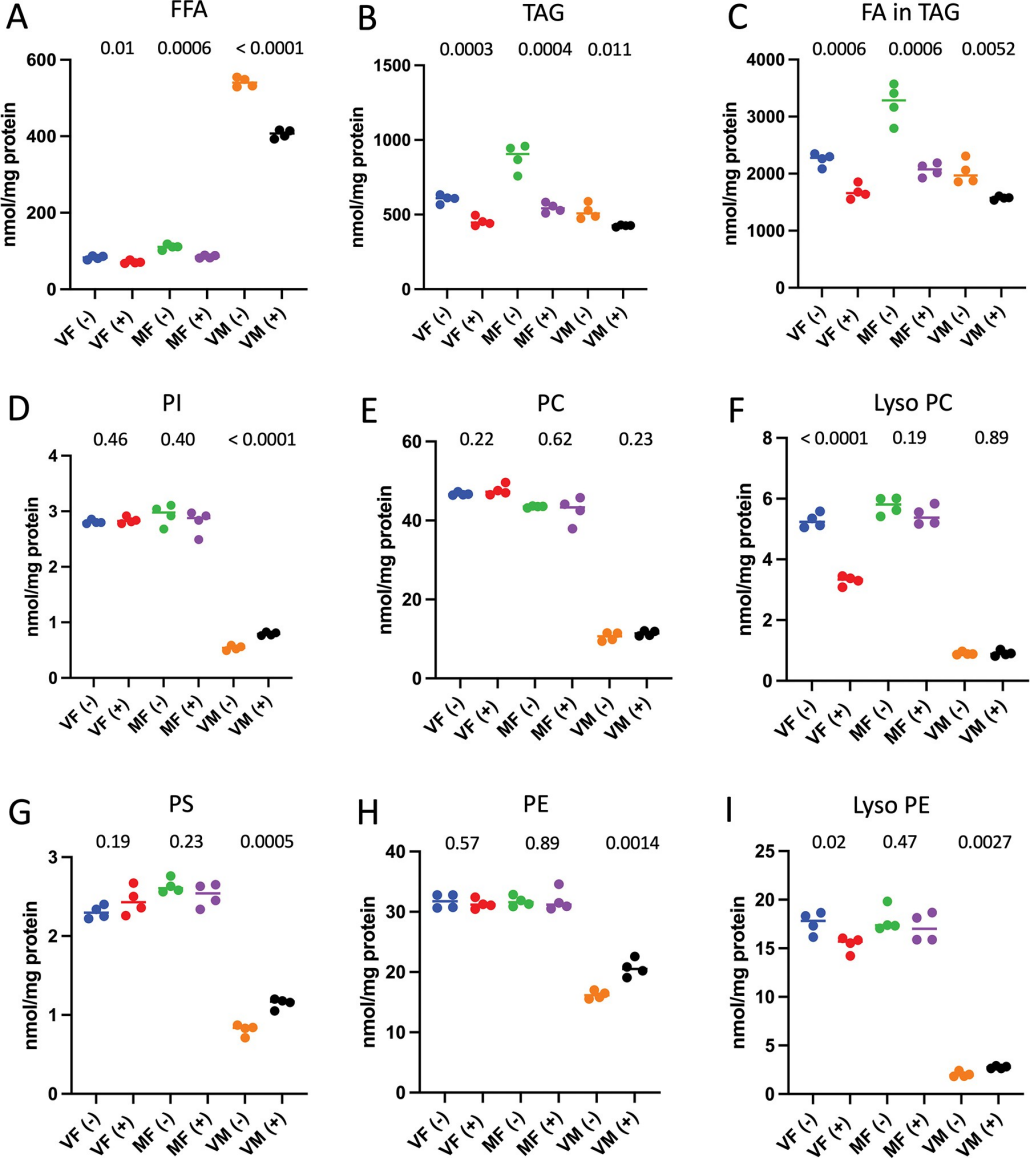
Lipidomics analysis of major lipid classes. The indicated lipid classes were quantified in flies after 12 days treatment with mifepristone and in no-drug controls. Four replicates of 10 flies each were assayed for each group, and the four replicate values are plotted with the mean. Each control group was compared to the corresponding drug-treated group using unpaired, two-sided t test, and the *p* value is presented at top of the plot; the p value for significance with one comparison is *p* < 0.05. A. Free fatty acids (FFA). B. Triglycerides (TAG). C. Fatty acids in triglycerides (FA in TAG). D. Phosphatidylinositol (PI). E. Phosphatidylcholine (PC). F. Lyso phosphatidylcholine (Lyso PC). G. Phosphatidylserine (PS). H. Phosphatidylethanolamine (PE). I. Lyso phosphatidylethanolamine (Lyso PE). VF, virgin females. MF, mated females. VM, virgin males. (-), no drug controls. (+), mifepristone treated (200μg/ml mifepristone).

**Fig 6:**
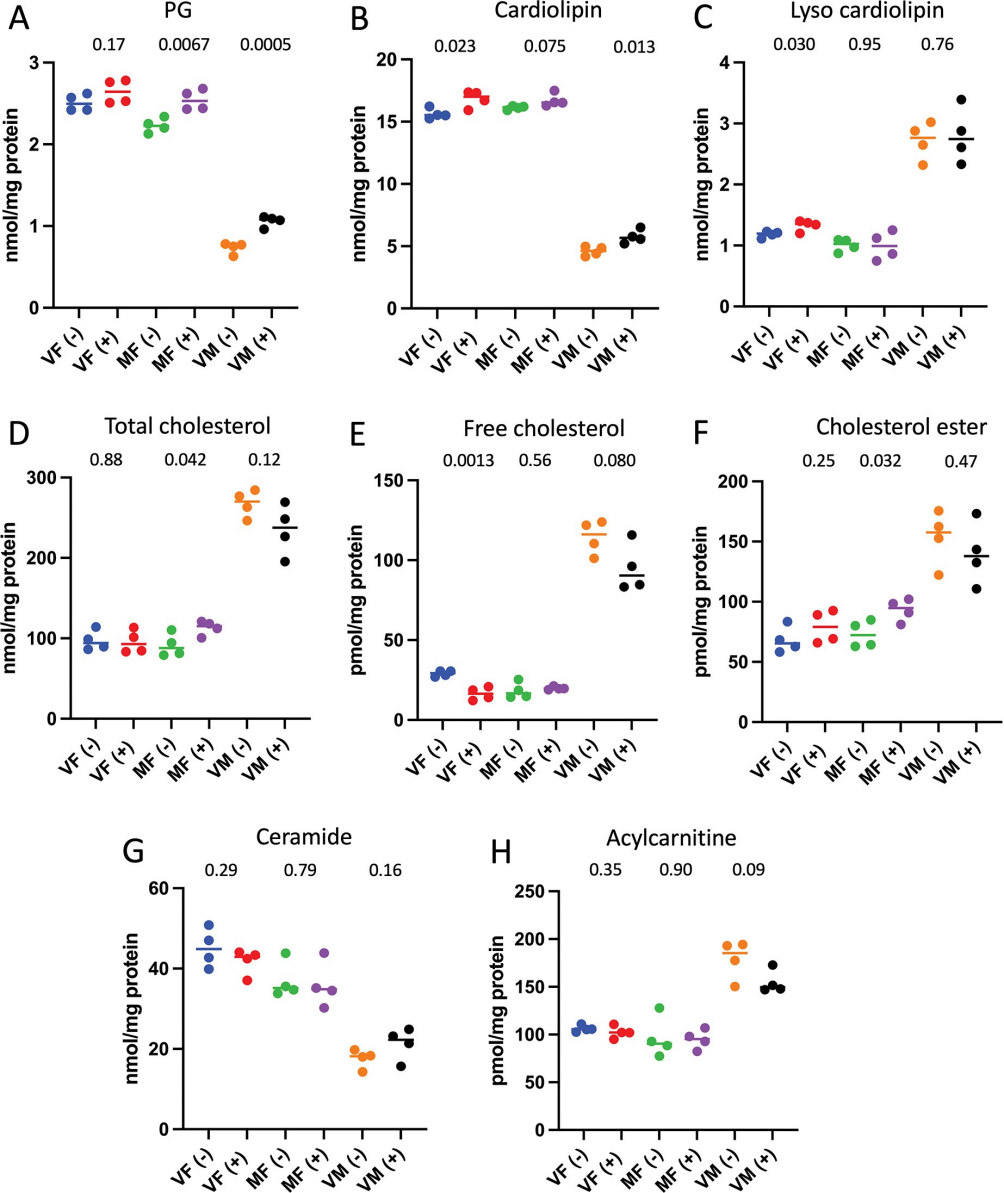
Lipidomics analysis of phosphatidylglycerol, cardiolipin, cholesterol and additional lipids. The indicated lipid classes were quantified in flies after 12 days treatment with mifepristone and in no-drug controls. Four replicates of 10 flies each were assayed for each group, and the four replicate values are plotted with the mean. Each control group was compared to the corresponding drug-treated group using unpaired, two-sided t test, and the p value is presented at top of the plot; the *p* value for significance with one comparison is *p* < 0.05. A. Phosphatidylglycerol (PG). Cardiolipin. C. Lyso cardiolipin. D. Total cholesterol. E. Free cholesterol. F. Cholesterol ester. G. Ceramide. H. Acylcarnitine. VF, virgin females. MF, mated females. VM, virgin males. (-), no drug controls. (+), mifepristone treated (200μg/ml mifepristone).

Whole-body, multidimensional mass spectrometry-based shotgun lipidomics was conducted on control and mifepristone-treated flies of the *w[1118]* control strain, and enabled quantification of 226 lipid species (S3 Table) [40–42]. Four replicates of 10 flies each were assayed for each of 6 groups: virgin males, virgin females and mated females, with and without mifepristone treatment. Measurements were generally highly consistent across replicates, and a significant remodeling of the lipidome by mifepristone was observed (Figs 5 and 6). Certain effects of mifepristone were observed for each of virgin females, mated females and virgin males, including decreased free fatty-acids (FFA; Fig 5A), decreased triglycerides (TAG; Fig 5B), and decreased fatty-acids (FA) in TAGs (Fig 5C). Several changes were unique to virgin females, including decreased lyso-phosphatidylcholine (Lyso PC; Fig 5F), decreased lyso-phosphatidylethanolamine (lyso PE; Fig 5I), increased lyso-cardiolipin (Fig 6C), and decreased free cholesterol (Fig 6E). In contrast, certain changes were significant only in mated females, including increased total cholesterol and cholesterol ester (Fig 6D and 6F). Several changes were unique to males, including increased phosphatidylinositol (Fig 5D), and increased phosphatidylserine and phosphatidylethanolamine (Fig 5G and 5H).

In addition to differences in the effect of mifepristone across groups, differences in baseline lipid levels were also observed across the groups. For example, relative to virgin females, mated females had relatively greater levels of FFA, TAG, and FA in TAG (Fig 5A–5C). Males exhibited a number of striking differences relative to both virgin females and mated females. For example, males had relatively greater abundance of FFA (Fig 5A), lyso cardiolipin (Fig 6C), total cholesterol, free cholesterol and cholesterol ester (Fig 6D–6F), and acylcarnitine (Fig 6H). In contrast, males had relatively lower abundance of PI, PC, lyso PC, PS, PE and lyso PE (Fig 5D–5I), PG and cardiolipin (Fig 6A and 6B), and ceramide (Fig 6G).

### Maternal *tudor[1]* mutation effect on life span and response to mifepristone

To begin to investigate the role of the germ line and egg laying in the effects of mifepristone, the *tudor[1]* mutation was analyzed. Maternal *tudor[1]* mutation results in female progeny that lack the germ-line and produce no eggs [30]. Because of this grandchildless phenotype, the *tudor[1]* mutation is not viable as a homozygous stock, and the *tudor[1]* bearing chromosomes have been maintained over balancer chromosomes at the stock centers for decades. As a consequence, background mutations accumulate on the *tudor[1]* bearing chromosomes, and homozygotes have extremely reduced viability. Even when *tudor[1]* is balanced over a *CyO* chromosome bearing a temperature-sensitive lethal mutation (BDSC stock #1786), and temperature shift is used to kill balanced flies during development, few homozygous *tudor[1]* flies are obtained, and these flies appear sickly. To overcome this limitation, two different strains bearing *tudor[1]* balanced over *CyO* were crossed to each other to generate abundant trans-heterozygous *tudor[1]* mutant females, as well as *tudor[1]/CyO* females, and each type of female was crossed to males of the *w[1118]* reference strain (crossing scheme presented in S2 Fig). The progeny of the *tudor[1]* homozygous mothers will lack the germ-line, whereas the progeny of *tudor[1]/CyO* mothers will be germ-line intact, and both groups of progeny will have the same genotype of *tudor[1]/+*.

**Fig 7:**
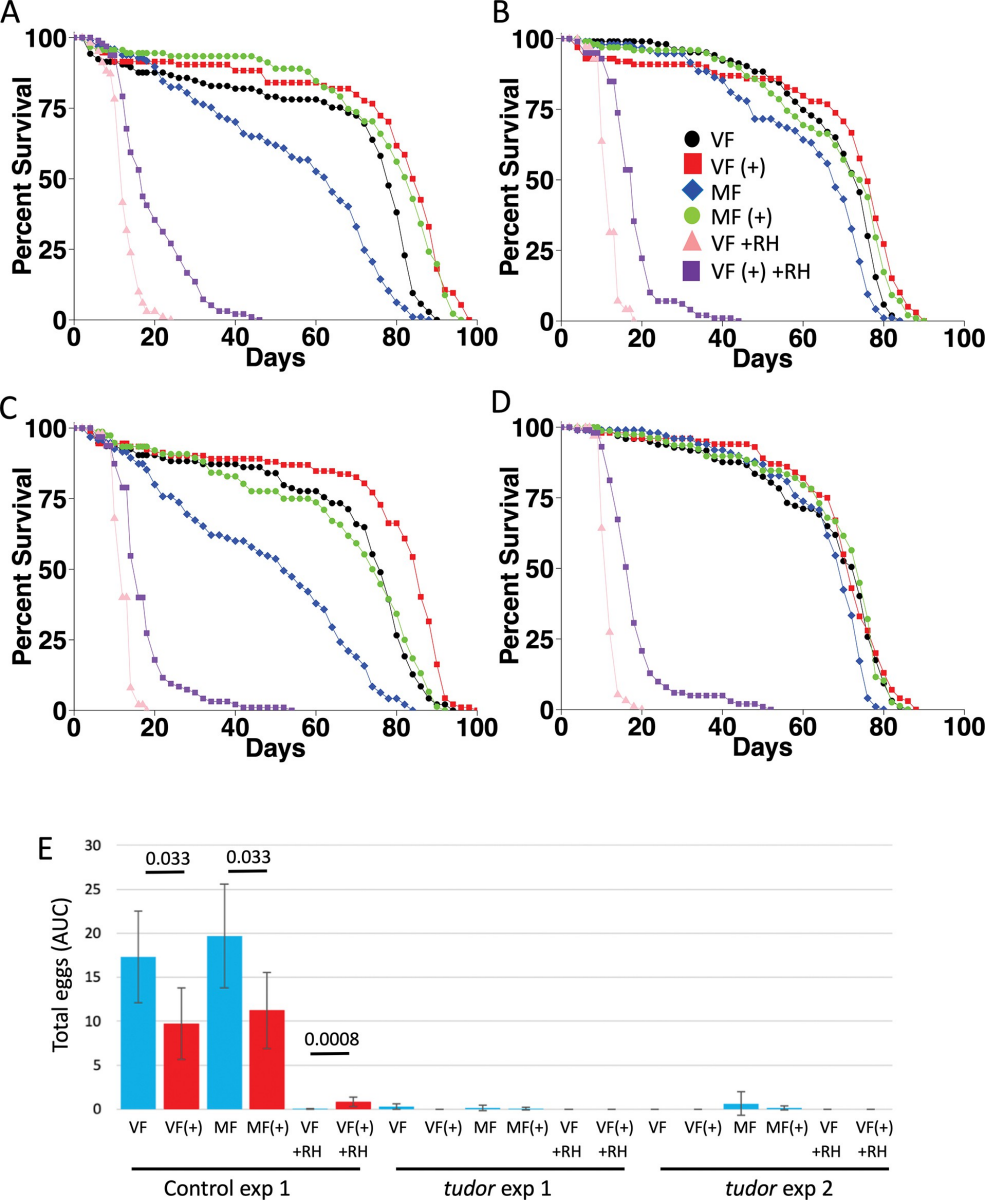
Maternal *tudor[1]* mutation effect on life span, egg laying and response to RH5849 and mifepristone. (A-D) Life span assays. A. Control mothers, experiment 1. B. *tudor[1]* mutant mothers, experiment 1. C. Control mothers, experiment 2. D. *tudor[1]* mutant mothers, experiment 2. VF, virgin female. MF, mated female. (+), 200μg/ml mifepristone. +RH, 1000μg/ml RH5849. Statistical summaries presented in Table 3. E. Egg laying assay. Egg counts were generated at days 8, 12, and 16, and area-under-curve analysis was used to estimate total eggs laid per fly. The statistical test is unpaired, two-sided t test, and the *p* value for significance with one comparison is *p* ≤ 0.05. The *p* value for pair-wise comparisons is presented above the plots. VF, virgin female. MF, mated female. (+), 200μg/ml mifepristone. +RH, 1000μg/ml RH5849.

Life span was assayed in virgin females and mated females, in presence and absence of mifepristone, in germ-line ablated females and genetically matched controls, in two replicate experiments (Fig 7A–7D), as well as in a third replicate of the germ-line ablated females (Table 3). COX-PHA revealed significant effects of mifepristone, mating, and maternal *tudor[1]* mutation, as well as significant interactions between mifepristone and mating, between mifepristone and maternal *tudor[1]* mutation, and between mating and maternal *tudor[1]* mutation (S4 Table). The negative life span effect of mating was reduced, but still significant, in the germ-line ablated females: Mating decreased life span in control females by -18% to -32%, and decreased life span in germ-line ablated females by -3% to -8% (Table 3). Similarly, the positive effect of mifepristone was reduced, but still significant, in the germ-line ablated females: Mifepristone increased life span in mated control females by +31% to +44%, and increased life span in mated germ-line ablated females by +6% to +10% (Table 3).

**Table 3:** Maternal *tudor[1]* mutation effect on life span and response to mifepristone and RH5849.

Experiment	Genotype (mothers)	Sex/status	Drug	N	Med	90% Mort	ΔMed (%)	*p*
1	*tud/+*	VF	-	105	78	84		
1	*tud/+*	VF	(+)	94	85	93	8.97	6.23E-9
1	*tud/+*	MF	-	97	64	79	-17.9	2.14E-9
1	*tud/+*	MF	(+)	91	84	92	31.4	9.10E-17
1	*tud/+*	VF	RH	101	12	16	-84.6	1.23E-33
1	*tud/+*	VF	RH (+)	96	17	32	41.7	9.58E-12
1	*tud/tud*	VF	-	103	74	80		
1	*tud/tud*	VF	(+)	99	76	84	2.70	0.0004
1	*tud/tud*	MF	-	95	68	76	-8.11	0.0008
1	*tud/tud*	MF	(+)	98	75	82	10.3	5.48E-6
1	*tud/tud*	VF	RH	99	12	14	-83.8	1.74E-42
1	*tud/tud*	VF	RH (+)	99	18	22	50.0	2.11E-18
2	*tud/+*	VF	-	94	76	86		
2	*tud/+*	VF	(+)	92	86	92	13.2	1.20E-7
2	*tud/+*	MF	-	95	52	74	-31.6	5.15E-13
2	*tud/+*	MF	(+)	76	75	88	44.2	2.08E-10
2	*tud/+*	VF	RH	100	12	14	-84.2	5.80E-31
2	*tud/+*	VF	RH (+)	95	16	24	33.3	7.77E-11
2	*tud/tud*	VF	-	97	74	80		
2	*tud/tud*	VF	(+)	100	72	82	-2.70	0.4473
2	*tud/tud*	MF	-	99	70	76	-5.41	0.0010
2	*tud/tud*	MF	(+)	78	74	81	5.71	7.68E-5
2	*tud/tud*	VF	RH	95	12	14	-83.8	7.58E-38
2	*tud/tud*	VF	RH (+)	101	18	24	50.0	2.03E-16
3	*tud/tud*	VF	-	78	72	79		
3	*tud/tud*	VF	(+)	92	76	86	5.56	0.0001
3	*tud/tud*	MF	-	97	70	75	-2.77	0.0014
3	*tud/tud*	MF	(+)	106	74	84	5.71	4.53E-8
3	*tud/tud*	VF	RH	96	12	16	-83.3	2.57E-25
3	*tud/tud*	VF	RH (+)	102	18	28	50.0	6.78E-18

(+), 200μg/ml mifepristone. RH, 1000μg/ml RH5849. In each experiment, MF (-) is compared to VF (-) to determine effect of mating, and for each sex/status, (+) is compared to (-) to determine effect of mifepristone, and RH (+) is compared to RH to determine effect of mifepristone in presence of RH. Statistical test is log-rank, and the p value for significance with three comparisons is p ≤ 0.0167.

RH5849 is a potent mimic of ecdysone [11, 43]. Our previous studies showed that 1000μg/ml RH5849 dramatically shortens life span in virgin females, and that this effect could be partially rescued by mifepristone [39]. This concentration of RH5849 was chosen because it was able to give a greater activation of an ecdysone-receptor-responsive transgenic reporter than was achieved with dietary ecdysone [39]. The previous study also showed that RH5849 significantly reduced food intake, and that mifepristone had no significant effect on food intake in the presence or absence of RH5849 [39]. Here the effects of RH5849 on life span were assayed, and found to be similar in the control and germ-line ablated females. RH5849 decreased life span in control virgin females by -84% to -85%, and decreased life span in germ-line ablated females by -83% to -84%. Mifepristone increased life span in RH5849-treated control virgins by +33% to +42%, and increased life span in RH5849-treated germ-line ablated females by +50% in each replicate experiment (Table 3). These data indicate that the ovary is not required for the negative effect of RH5849 on life span, nor is the ovary required for mifepristone to rescue the negative effect of RH5849 on life span.

**Fig 8:**
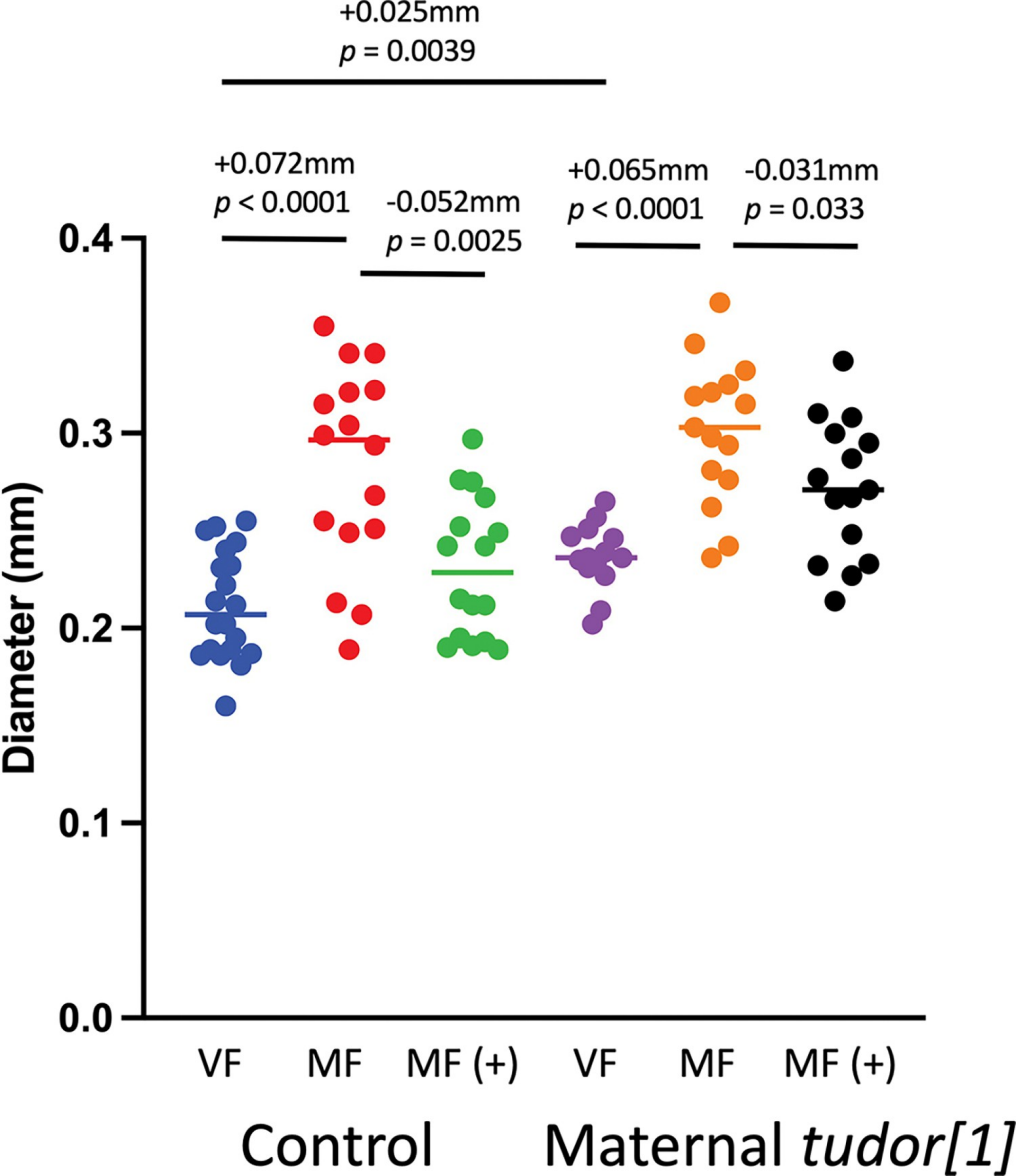
Maternal *tudor[1]* mutation effect on maximum midgut diameter. The statistical test is unpaired, two-sided t test, and the *p* value for significance with two comparisons is *p* ≤ 0.025. The change in mean maximum midgut diameter between samples and the *p* value for significance is presented above the plots. VF, virgin female. MF, mated female. (+), 200μg/ml mifepristone.

Egg laying was assayed in 12 groups: virgin females, mated females, and virgin females treated with RH5849, in presence and absence of mifepristone, for both control and germ-line ablated females. Eggs were quantified at days 8, 12, and 16 of drug treatment, in 5 replicate vials of 20 flies each, for each of the 12 groups (S3 Fig). Total eggs laid was estimated using area-under-curve, and consistent with the experiments presented above, mifepristone significantly decreased egg production in both virgin females and mated females in control flies (Fig 7E). As expected, eggs were essentially absent in the germ-line ablated female groups. Zero eggs were found in 73/80 vials at each time point (S3 Fig), and no eggs or differentiated ovary structures were detected in >50 dissected germ-line ablated females (see midgut analysis, Fig 8). Eggs were detected in 7/80 vials of germ-line ablated females (S3 Fig); however, we conclude these eggs resulted from a single contaminating control fly in each of the 7 vials, as the number of flies produced was approximately 1/20 of that observed in vials containing 20 control flies (see Discussion).

Midgut maximum diameter was assayed in virgin females, mated females, and mated females treated with mifepristone, in control and germ-line ablated females (Fig 8). As expected, midgut diameter was increased by mating, and decreased by mifepristone in control females (Fig 8). In the germ-line ablated females, mating produced an increase in midgut size of similar magnitude to that observed in controls (Fig 8). In contrast, mifepristone produced a smaller decrease in midgut size in germ-line ablated females than in control females, and this change was not significant after control for multiple comparisons (Fig 8).

### Effect of mifepristone and genotype on life span and egg production in virgin females

As shown above, mifepristone increases life span in virgin females of the control genotype for the *tudor[1]* experiments by +9% and +13%, respectively (Fig 7A and 7C; Table 3), and this genotype is wild-type for the *white* gene. Similarly, our previous studies of a long-lived genotype (progeny of *w[1118]* x *yw;ElavGS* cross) also found robust life span extension by mifepristone in virgin flies, ranging from approximately +15% to +30% [18, 21]; this genotype is null mutant for *white*, but bears a single copy of the *mini-white[+]* marker transgene, and so is at least partially *white[+]*. In contrast, as shown above (Figs 1 and 2; Table 1), mifepristone did not increase life span in virgin females of the *white* homozygous null mutant strain *w[1118]*.

**Fig 9:**
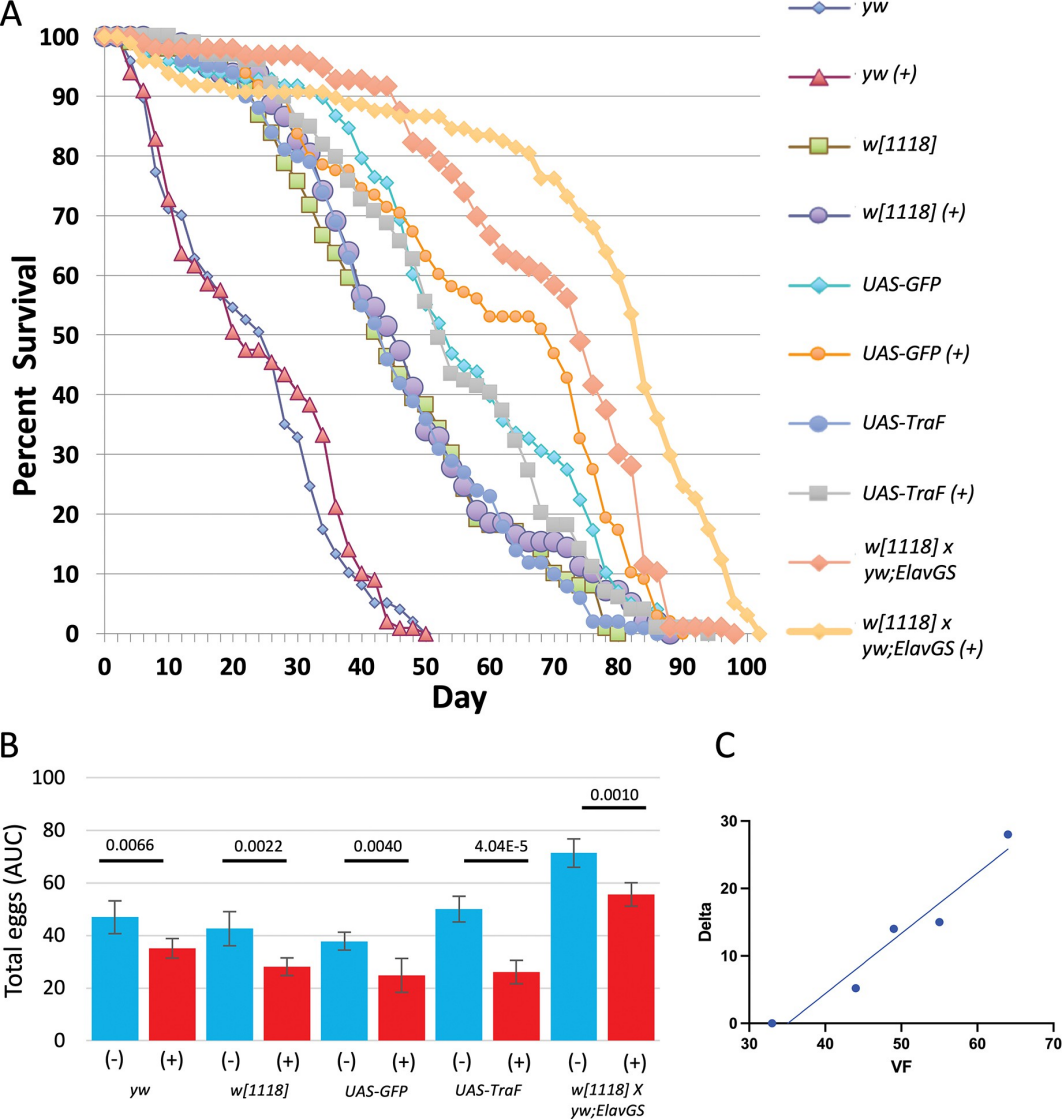
*White* gene mutation effect on life span and egg laying. A. Virgin females of the indicated genotypes were assayed for life span in absence and presence (+) of 200μg/ml mifepristone. Statistical summary including replicate experiment presented in Table 4. B. Egg laying assay. Virgin females of the indicated genotypes were assayed for egg production every other day from days 2 to 34 (trajectories presented in S4 Fig). Area-under-curve analysis was used to estimate total eggs laid per fly. The statistical test is unpaired, two-sided t test, and the *p* value for significance with one comparison is *p* ≤ 0.05. The *p* value for pair-wise comparisons is presented above the plots. (+), 200μg/ml mifepristone. C. Effect of mifepristone on virgin female life span across strains. The average virgin female (VF) life span for the 5 strains is plotted on the X axis, in relation to the average change in life span caused by mifepristone (Delta) on the Y axis; negative life span effects of mifepristone were treated as zero. Linear regression R[2] = 0.94.

The lack of response of *w[1118]* virgin females to mifepristone might be due to the lack of *white* gene function, or might be due to some other mutation in the highly-inbred *w[1118]* genetic background. To begin to address these questions, two different P element insertions bearing the *mini-white[+]* marker transgene were backcrossed into the *w[1118]* genetic background for 9 generations, to ask if this would be sufficient to confer a response to mifepristone in virgin females. These two resulting strains (UAS-Tra and UAS-GFP) were assayed for virgin female life span in the presence and absence of mifepristone, alongside the *w[1118]* strain, the short-lived *yw* strain, and the long-lived genotype (progeny of *w[1118]* x *yw;ElavGS* cross) that is known to give robust response to mifepristone in virgin females, in two replicate experiments (Fig 9; Table 4).

**Table 4:** Effect of *mini-white[+]* on virgin female response to mifepristone.

Experiment	Genotype	Sex/status	Drug	N	Med	90% Mort	ΔMed (%)	*p*
1	*yw*	VF	-	99	32	42		
1	*yw*	VF	(+)	96	32	47	0.00	0.4566
1	*w[1118]*	VF	-	100	36	58		
1	*w[1118]*	VF	(+)	103	38	64	5.56	0.3270
1	*UAS-GFP*	VF	-	97	48	80		
1	*UAS-GFP*	VF	(+)	98	46	72	-4.00	0.0513
1	*UAS-tra*	VF	-	98	46	72		
1	*UAS-tra*	VF	(+)	98	50	74	8.70	0.2678
1	*w[1118] X yw;ElavGS*	VF	-	96	56	79		
1	*w[1118] X yw;ElavGS*	VF	(+)	93	80	92	42.9	1.29E-13
2	*yw*	VF	-	97	24	37		
2	*yw*	VF	(+)	99	20	38	-16.7	0.4169
2	*w[1118]*	VF	-	99	42	68		
2	*w[1118]*	VF	(+)	97	44	75	4.76	0.2953
2	*UAS-GFP*	VF	-	98	52	77		
2	*UAS-GFP*	VF	(+)	98	68	81	30.8	0.1054
2	*UAS-tra*	VF	-	100	42	68		
2	*UAS-tra*	VF	(+)	99	50	76	19.0	0.0018
2	*w[1118] X yw;ElavGS*	VF	-	96	72	85		
2	*w[1118] X yw;ElavGS*	VF	(+)	97	82	96	13.9	1.08E-6

(+), 200μg/ml mifepristone. In each experiment, for each genotype, (+) is compared to (-) to determine effect of mifepristone. The statistical test is log-rank, and the *p* value for significance with one comparison is *p* ≤ 0.05.

COX-PHA analysis of virgin female life span of the *w[1118]* strain and the two backcrossed *mini-white[+]* strains, in presence and absence of mifepristone, revealed no significant effect of mifepristone, a significant effect of *mini-white[+]*, and no significant interaction between *mini-white[+]* and mifepristone (S5 Table). Therefore, whereas *mini-white[+]* did confer some increase in virgin female life span relative to *w[1118]*, it did not confer a consistent life span response to mifepristone in virgin females. In addition, no increase in virgin female life span in response to mifepristone was observed for virgin females of the short-lived *yw* strain. As expected, mifepristone produced a robust increase in life span in virgin females of the long-lived genotype (progeny of *w[1118]* x *yw;ElavGS* cross)(Table 4). In previous studies, the effect of mifepristone on life span in mated females across different genotypes was found to be roughly proportional to the starting virgin life span of the strain, with longer-lived strains showing a greater magnitude response [19]. Here, a similar trend was observed for the effect of mifepristone on virgin life span across strains, where the longest-lived starting strain shows the largest response (Fig 9C).

To investigate the effect of mifepristone and genotype on egg production in virgin females, the same 5 strains were assayed for number of eggs laid per day, every other day from day 2 to day 34, in presence and absence of mifepristone (S4 Fig). Area-under-curve analysis was used to estimate total egg production (Fig 9B). Mifepristone significantly reduced egg production in each strain, demonstrating that mifepristone has physiological effects in each strain, and indicating that decreased egg production is not sufficient for mifepristone to confer increased life span in virgin females.

## Discussion

A previous targeted metabolomics analysis of the effects of mating and mifepristone found that mating increases the abundance of numerous AAs and AA metabolites, as well as three lipids, and that these changes are reversed by mifepristone [20]. In addition, mifepristone increased survival of female flies fed a HFD, without decreasing food intake. These results suggested that AA metabolism and/or lipid metabolism may limit life span in the mated female fly. The drug etomoxir inhibits carnitine palmitoyltransferase I (CPT I), which is the rate-limiting enzyme for transport of long-chain FAs into the mitochondria [44]. Similar to mifepristone, etomoxir was found to increase life span in female flies, but not males, thereby supporting the idea that increased lipid metabolism limits female Drosophila life span [39]. Here, experiments were undertaken to further investigate the possible role of lipid metabolism in the life span effects of mifepristone.

### Dhr96 and lipidomics

The Drosophila Dhr96 hormone receptor is related to mammalian hormone receptors that regulate lipid metabolism, including the vitamin D receptor (VDR/ NR1I1) and the LXR receptor (NR1H) [29, 34]. Consistent with this relationship, Dhr96 functions specifically in the Drosophila midgut to promote lipid uptake and metabolism, and *Dhr96[1]* null mutant flies have reduced whole-body levels of TAG [33, 34]. Recent network modeling using ChIP-Seq and protein-protein interaction data suggests that *Dhr96* regulates TAG metabolism in part through regulation of mitochondrial genes to stimulate lipid consumption and mitochondrial respiration [45].

Here the *Dhr96[1]* null mutation was found to increase life span in female Drosophila, consistent with the idea that increased intestinal lipid uptake and/or metabolism may limit life span in females. The *Dhr96[1]* mutation reduced, but did not eliminate, the positive effect of mifepristone on mated female life span, suggesting that *Dhr96[1]* and mifepristone may act in part through a common mechanism of reducing intestinal lipid metabolism and TAG levels. Indeed, Oil Red O staining of lipids in midgut tissue indicates reduced midgut lipid content due to mifepristone treatment [21, 46]. Consistent with this idea, lipidomics analysis revealed that mifepristone reduced whole-body levels of TAG, as well as FFA and FA in TAG, in each of virgin females, mated females and males. Moreover, *Dhr96[1]* mutation is reported to increase levels of total cholesterol in Drosophila larvae and adults [34, 35], and here the lipidomics analysis showed increased total cholesterol and cholesterol ester levels in mated females treated with mifepristone. Taken together, these results suggest that Dhr96 might be a direct or indirect target of inhibition by mifepristone. Conceivably, mifepristone might compete with cholesterol for binding to Dhr96, thereby directly reducing Dhr96 activity. In addition, because Dhr96 functions in the midgut, and mifepristone reduces midgut maximum diameter in mated females, mifepristone might indirectly reduce Dhr96 activity in mated females by reducing the volume of tissue in which Dhr96 is expressed.

Here the *Dhr96[1]* mutation was assayed in the *w[1118]* reference strain background, and we note that the negative effect of mating on life span in the *w[1118]* control strain varied across replicate experiments, from -10.5% to -27.9% (Table 1). The COX-PH analysis indicated a significant life span interaction between *Dhr96[1]* mutation and mating. Consistent with that result, 3 out of 4 assays of the *w[1118]* control strain showed a significant negative effect of mating, with an average decrease of -21%, whereas 3 out of 4 assays of the *Dhr96[1]* mutant strain showed a significant negative effect of mating, with an average decrease of -37.5%. Therefore, the negative effect of mating was on average greater in the *Dhr96[1]* mutant flies relative to the *w[1118]* controls, but variability in the magnitude of effect was observed across replicates for both groups. The reason(s) for this variability in the negative effect of mating is unknown at this time, and underscores the importance of using replicate assays when comparing different genotypes.

Previous studies report that Dhr96 activates expression of numerous transporters and lipid metabolism regulators in the midgut, including several Niemann-Pick-related genes and the LipA-related enzyme Magro, which is a TAG lipase and cholesterol esterase [33]. Moreover, RNAi knock-down of *magro* was sufficient to recapitulate several aspects of the *Dhr96[1]* mutant phenotype, including reduced TAG levels and increased cholesterol [33, 34]. In the future it may be of interest to test the loss of function of *magro* and other specific Dhr96 targets for ability to mimic the benefits of mifepristone, including increased life span. The *Dhr96[1]* mutation was previously reported to slightly decrease life span in a single assay of mated females, when backcrossed into a *w[1118]*-*Dahomy* background [37], whereas here increased life span in mated females was observed. This difference in results may be due to the different genetic background relative to the *w[1118]* reference strain used here. Mifepristone was recently reported to inhibit an *estrogen-related receptor* (*ERR*) transgenic reporter in adult Drosophila, and to reduce lipid staining and *magro* RNA levels in the midgut in flies of undefined sex [46], and in the future it may be of interest to test loss of function of Drosophila *ERR* for ability to mimic the benefits of mifepristone. Finally, naturally-occurring genetic variation in the Drosophila gene *Eip75B* has been found to be associated with adult life span and fecundity [47, 48], and experimental knock-down studies in adult flies show that *Eip75B* is required for midgut hypertrophy in response to mating [11, 12, 15], for normal adult life span and egg production [48, 49], and for normal sugar tolerance and expression of de novo lipogenesis genes [50]; in the future it may be of interest to determine if adult-specific knock-down of *Eip75B* might affect the response to mifepristone.

Similar to the ability of mifepristone to reduce whole-body TAG levels in adult Drosophila, mifepristone treatment also reduces serum TAG levels in mice and humans [5, 7, 46], consistent with a possible conservation of mifepristone mechanisms across species. Drosophila Dhr96 is related to the mammalian vitamin D receptor (VDR/NR1I1), as well as to the mammalian LXR receptor (NR1H) [29, 34]. Mice mutant for VDR are protected from HFD-induced obesity, inflammation and liver disease, and show decreased lipid uptake, decreased lipase activity, and a trend towards increased food intake [51], each reminiscent of mifepristone effects in Drosophila. Mammalian LXRa binds oxysterol ligands and promotes the modification and clearance of excess sterols [52]. In addition, LXRa maintains proper triglyceride (TAG) levels, in part through activation of SREBP which in turn regulates fat synthesis [53]. In the future, it may be of interest to ask if knock-down of VDR and/or LXRa activity in mammals will reduce the beneficial metabolic effects of mifepristone in mammals.

### Lipidomics and effects of sex and mating status

In the *w[1118]* strain, mating decreases female life span, and mifepristone increases life span of mated females. Correspondingly, mating caused increased levels of TAG, FFA, and FA in TAG in females, which was in turn reduced by mifepristone treatment, consistent with the idea that reduction of lipid uptake and metabolism may be part of the mechanism for mifepristone life span increase in mated females. Indeed, the two most abundant lipid groups, TAG, and FA in TAG, exhibited the strongest inverse correlation with life span, in that they underwent the largest fold increase due to mating, and the largest fold decrease due to mifepristone. However, mifepristone also produced smaller fold decreases in TAG, FFA, and FA in TAG in virgin females and in virgin males, but did not increase life span in those groups, consistent with at least partially different mechanisms for mifepristone life span regulation depending on sex and mating status.

Previous studies report some variable results regarding the relationship between whole-body lipid levels and life span. Mating has been reported to reduce TAG levels in females of an *Oregon-R* strain [Koliada et al., 54], whereas here we find mating causes increased levels of TAG in *w[1118]* strain females; conceivably this difference in results is related to the use of different genotypes. Recently, Shaposhnikov et al. investigated several life span-extending interventions, including *E(z)* gene mutation, phytochemicals, reduced temperature, and reduced light exposure, and found that life span increase was not associated with total lipid levels [Shaposhnikov et al., 55]. In contrast, when *Canton-S* strain life span was altered by nutritional geometry, the longest lived flies had greater lipid content [Lee et al., 56], and in one study of Drosophila lines selected for postponed senescence, the long-lived flies had less lipid content than controls [Nasiri et al., 57]. These differences in results may be related to the specific type of life span intervention employed, as well as the use of different genotypes. As discussed above, at least for mated females, we find that interventions that reduce lipid uptake and/or metabolism, including mifepristone, etomoxir, and *Dhr96[1]* mutation, are associated with increased life span.

A number of striking differences in baseline lipid levels were observed in males relative to females, including both increases and decreases. For example, Drosophila males had decreased levels of total TAG, and decreased levels of mono-unsaturated and poly-unsaturated lyso PC relative to Drosophila females, consistent with previous reports [14, 58, 59]. Striking differences in circulating lipid levels are also observed when comparing men to women [60–62].

### Maternal *tudor[1]* mutation and effect of germ-line ablation on life span

To ask whether altered egg production is required for the life span effects of mating and mifepristone, the maternal *tudor[1]* mutation was used to create female flies that lack the germ-line [30]. The complete absence of eggs and differentiated ovary structures was confirmed by dissection of >50 females. The germ-line ablated females were maintained as adults with 20 flies per vial, and 73/80 vials produced zero eggs over multiple time points. In contrast, 7/80 vials produced multiple eggs over one or more time points. We conclude these eggs resulted from a single contaminating control fly in each of the 7 vials, because the number of eggs produced was roughly 1/20 of those produced by a vial of 20 control flies. This slight contamination would be consistent with the mis-scoring of one or two maternal *Cy* flies as non-*Cy* in step two of the crossing scheme (S2 Fig). A final contamination frequency of 7/1593 flies, or 0.4%, is not expected to significantly affect results.

In the germ-line ablated females, both the negative effect of mating on life span, and the positive effect of mifepristone on mated female life span were reduced, but still significant. These results suggest that a possible negative effect of egg production or egg laying might contribute to the life span effects of mating and mifepristone, but is not essential. Consistent with this conclusion, previous studies report that reducing egg production using sterile mutants can sometimes increase Drosophila life span, and also that life span and egg production can sometimes be uncoupled [63, 64]. A previous study of maternal *tudor[1]* mutation effect on virgin females reported decreased life span [65], and consistent with that observation, here maternal *tudor[1]* mutation was also found to be associated with slight decreases in median life span in virgin females. Our previous study of maternal *tudor[1]* mutation effect in mated females found no consistent change in life span, where mating was conducted by exposing newly-eclosed females to newly-eclosed male siblings over 24 hours [66]. In contrast, here the maternal *tudor[1]* mutation resulted in increased life span in mated females. This difference may be due to the more intensive mating protocol employed here, where newly-eclosed females were mated to young, mature *w[1118]* strain males for 48 hours. Consistent with this conclusion, our previous studies indicated that 48 hours mating reduced female life span to a greater extent than did 24 hours mating [39], and that the more intensive mating protocol yields shorter mated female life spans and greater increases in life span upon mifepristone treatment [19].

### Effects of mating and mifepristone on maximum midgut diameter

The ability of mating and male SP to cause midgut hypertrophy in the mated Drosophila female has been well characterized [11–13, 15, 21, 67], but the potential link between midgut hypertrophy and decreased life span remains unclear. The opposing effects of mating and mifepristone on maximum midgut diameter have been consistently observed across each of 4 control strains tested, including the SREBP reporter strain [21], the progeny of the *w[1118]* x *yw;ElavGS* cross [39], the *w[1118]* reference strain [21] (Fig 4), and the control genotype for the maternal *tudor[1]* flies (Fig 8). Here the effects mating and mifepristone on maximum midgut diameter were assayed in flies with maternal *tudor[1]* mutation and *Dhr96[1]* null mutation, and compared with the changes in life span.

As discussed above, the effects of mating and mifepristone on life span were reduced, but still significant, in the *tudor[1]* females with ablated germ-line. Some correlation was observed between these effects on life span and changes in maximum midgut diameter, in that the effect of mating on maximum midgut diameter also appeared reduced in the germ-line ablated females. In control females, mating caused an increase in maximum midgut diameter of +0.072mm, and in germ-line ablated females mating produced a relatively smaller increase of +0.065mm. Similarly, in control mated females, mifepristone decreased maximum midgut diameter by -0.053mm, whereas in mated germ-line ablated females this decrease was -0.031mm, and this change was not significant after controls for multiple comparisons. Therefore, the reduced effect of mating and mifepristone on life span in germ-line ablated females was generally correlated with a reduced effect of mating and mifepristone on maximum midgut diameter.

Some correlation between life span and maximum midgut diameter was also observed for the effects of the *Dhr96[1]* null mutation. The *Dhr96[1]* mutation reduced, but did not eliminate the life span effects of mating and mifepristone. Consistent with this, the effect of mifepristone on maximum midgut diameter was reduced in mated females of the *Dhr96[1]* strain. In the *w[1118]* control strain, mifepristone reduced maximum midgut diameter of mated females by -0.051mm, and in *Dhr96[1]* strain mifepristone reduced maximum midgut diameter by -0.031mm, and this was not significant after controls for multiple comparisons. Therefore, the reduced effect of mifepristone on life span in *Dhr96[1]* null mutant mated females correlated with a reduced effect of mifepristone on maximum midgut diameter.

In contrast, a novel pattern was observed for the effects of mating in the *Dhr96[1]* mutant flies. In *w[1118]* control flies, mating caused an increase in maximum midgut diameter of +0.037mm, and this correlated with decreased life span, as expected. However, in the *Dhr96[1]* null mutant females, virgin females exhibited a maximum midgut diameter that was increased by +0.052mm relative to the *w[1118]* virgin female controls, and this was not further altered by mating after controls for multiple comparisons. As discussed above, the *Dhr96[1]* mutation is associated with increased life span in virgin females relative to the *w[1118]* controls. Therefore, the increased maximum midgut diameter observed in virgin females of the *Dhr96[1]* strain does not correlate with a reduced life span. One possible interpretation of the increased maximum midgut diameter observed in the *Dhr96[1]* virgin females is that this represents a constitutive mating-like state. Alternatively, the increased maximum midgut diameter observed in the *Dhr96[1]* virgin females might represent some alternative mechanism for hypertrophy, perhaps explaining the lack of reduced life span. Notably, Obniski et al reported that *Dhr96[1]* mutation caused reduced abundance of enteroendocrine cells in the adult midgut, as well as increased expression of Delta and the Notch extracellular domain [68]. Because Delta/Notch signaling regulates ISC maintenance, proliferation and daughter cell differentiation [69–71], these increases in Delta/Notch expression might be related to the increase in maximum midgut diameter observed in virgin females with *Dhr96[1]* mutation. In the future, it may be of interest to further explore this phenomenon using cell biology approaches, such as quantification of cell size and proliferation.

### *w[1118]* and effects of genetic background

As discussed above, mifepristone increases life span and reduces maximum midgut diameter in mated females from multiple strains, including the *w[1118]* reference strain. Mifepristone also increases life span in virgin females of multiple strains, including progeny of the *w[1118]* x *yw;ElavGS* cross and the *Canton S* strain [19], but does not increase life span in virgin females of the *w[1118]* reference strain. The lack of mifepristone effect in *w[1118]* virgin females might be due to the *white* gene mutation, or to some other aspect of the *w[1118]* reference strain genetic background. Here, introduction of *mini-white[+]* transgenes increased baseline life span, but did not confer a response to mifepristone in virgin females. One possible interpretation of this result is that is it indeed the lack of *white* gene function that prevents a response to mifepristone in the *w[1118]* strain, but the *mini-white[+]* transgenes do not produce sufficient *white* gene function to enable a response to mifepristone. One way to address this in the future might be to test transgenes containing more extensive *white* gene sequences. The other possible interpretation of these results is that some other feature of the highly-inbred *w[1118]* genetic background limits the response to mifepristone in virgin females, such as possible background mutations that limit life span for a novel reason that does not respond to mifepristone. Consistent with the idea that background mutations that reduce life span might also limit response to mifepristone, for both mated females [19], and virgin females (Fig 9C), comparison across strains suggests that the longest-lived starting strains tend to show the largest responses to mifepristone.

### Summary and implications

The analysis of germ-line ablated female flies indicated that altered egg laying might contribute to the life span effects of mifepristone, but was neither strictly necessary nor sufficient. The reduced effect of mifepristone in *Dhr96[1]* mutant mated females, and the general correlation of mifepristone life span effects with whole-body TAG lipid abundance, supports the hypothesis that mifepristone may increase life span by reducing lipid uptake and/or metabolism. The ability of mifepristone to reduce maximum midgut diameter in mated female Drosophila is also generally consistent with reduced lipid uptake and/or metabolism. The ability of mifepristone to increase life span in virgin females of some Drosophila strains and not others indicates that mifepristone may increase life span in virgin females and mated females through partly different mechanisms. Interestingly, altered lipid metabolism is involved in the decreased life span observed in mated *C*. *elegans* [72, 73], and mifepristone can increase life span of mated, but not virgin, *C*. *elegans* [20], consistent with possible conservation of one or more mifepristone targets.

The effects of mifepristone on lipid levels and midgut size in Drosophila suggests some similarities to mammals. For example, mifepristone decreased serum TAG levels in male and female mice and humans [7, 46]. Recently, high dietary fructose was found to increase mouse gut size [74], and interestingly, mifepristone prevents insulin resistance and lipid abnormalities caused by a high fructose diet in mice [75]. There also appear to be some similarities with regard to sex bias. Women have greater prevalence of obesity, late-age colon cancer, and gastrointestinal (GI) disorders than men, including GI disorders associated with pregnancy [76–78]. In female mammals, lactation is associated with dramatic remodeling and growth of the intestine [79, 80], reminiscent of mating-induced midgut hypertrophy in female Drosophila. Taken together, the results with Drosophila provide additional insight into possible mechanisms for the anti-obesity and anti-diabetes effects of mifepristone observed in mice and humans, including the potential implication of Dhr96-related factors such as VDR and LXRa.

## Materials and methods

### *Drosophila* strains, culture, drug treatments and life span assay

*Drosophila melanogaster* flies were cultured and life span assays conducted in Percival brand incubators, at 25°C, 12:12 h light-dark cycle, at 65–80% humidity, using a standard agar/dextrose/corn meal/yeast media [81]. The media recipe is: 105 g dextrose, 7.5 g agar, 26 g yeast, 50 g cornmeal, 1 l purified H2O, boil for 30 min with constant agitation, then add 1.7 g tegosept (Genesee Scientific #20–259) dissolved in 8.5 ml 95% ethanol and 1.9 ml propionic acid (99%, Mallinckrodt Baker). To generate the high-fat diet (HFD), warm liquid media was supplemented with the indicated concentration of coconut oil (organic triple-filtered coconut oil, Trader Joes® brand), mixed for 2 minutes with a hand-held motorized blender, and then aliquoted to vials and allowed to cool. The strain *w[1118]; UAS-2xEGFP[m5B29]* is as previously described [82], abbreviated here as *UAS-GFP*. Several Drosophila strains were obtained from the Bloomington Drosophila Stock Center, including: strain *w[1118]; P{UAS-tra*.*F}20J7* (BDSC#4590), abbreviated here as *UAS-TraF*, strain *y[1] w[1]* (BDSC#1495), abbreviated here as *yw*, strain *vas*^*1*^ *cn*^*1*^ *tud*^*1*^ *bw*^*1*^ *speck*^*1*^*/CyO* (BDSC#1735), strain *tud*^*1*^ *bw*^*1*^ *speck*^*1*^*/CyO*, *l(2)DTS5131* (BDSC#1786), strain y[1] w[*]; P{w[+mC] = elav-Switch.O}GSG301 (BDSC#43642), abbreviated here as *yw;ElavGS*, and strain *TI{TI}Hr961* (BDSC#76592). The *TI{TI}Hr961* strain was backcrossed 9 generations to *w[1118]* strain, by following the *3xP3-eGFP* marker (S2A–S2C Fig), and the resultant homozygous strain is referred to here as *Dhr96[1]*. The *w[1118]* strain is the isogenized version (*w[1118]-iso; 2-iso; 3-iso*). The *w[1118]* strain was previously cured of Wolbachia by three generations treatment with doxycycline, with confirmation using PCR and Wolbachia-specific primers [83]. To generate flies for life span assays, female progeny were collected as virgins over 24 hours. These flies were either assayed as virgins, or were mated for 48 hours to young (1–2 weeks of age) *w[1118]* strain males at a ratio of 20 males to 20 females. After mating the males were removed, and the virgin female and mated female flies were maintained in culture vials with 20 flies per vial, in the presence/absence of drug, as indicated. Drugs were administered as previously described [81, 84], by applying 100μl of 10X stock solution in water, or 50μl of 20X stock solution in ethanol, evenly to the surface of the vial, and allowing to absorb and dry overnight. Final concentration of drug in the media was calculated based on absorption into the top ~1ml of media, as determined by dye-absorption controls [81, 84]; control vials received equal volume of water or ethanol vehicle, and all vials were allowed to dry overnight. Mifepristone (RU486) was obtained from Sigma-Aldrich (cat. #M8046), and flies were treated with 200μg/ml final concentration in the media; this concentration was determined to be optimal for life span extension in mated females by titrations in previous studies [18, 19]. Methoprene was obtained from Cayman Chemical (cat#16807), and flies were treated with 100μg/ml final concentration in the media. RH5849 (1,2-dibenzoyl-1-tert-butylhydrazine) was obtained from AABLOCKS (cat#AA00832D), and flies were treated with 1000μg/ml final concentration in the media. All flies were transferred to fresh vials every other day, and number of dead flies recorded. Median life span, percent change in median, log-rank *p* values and COX proportional hazards analyses were conducted using R statistical environment [85]. Log-rank analysis was corrected for multiple comparisons using Bonferroni correction, and the *p* value for significance at 5% error rate is indicated in the figure legends. The *p* value for significance is calculated as 0.05 divided by the number of comparisons used for each specific experiment.

### Midgut measurements

Virgin and mated female flies were generated as described above, the males were removed, and then the females were maintained in culture vials in the presence or absence of drug for 12 days. Maximum midgut diameter was assayed as previously described [21]. Briefly, midguts were dissected in PBS in groups of 5 flies at a time, and immediately mounted on slides with coverslip spaced using double-stick tape [86]. Visible light images were generated and analyzed using Image J. The maximum diameter region of each midgut sample was estimated by inspection, multiple measurements in that region were generated using Image J, and the largest value was used for analysis. Unpaired, two-sided t tests were conducted using Prism 9, and any outliers were identified using Grubbs test. Multiple comparisons were controlled using Bonferroni correction, and the *p* value for significance at 5% error rate is indicated in the figure legends.

### Egg laying assays

Virgin and mated flies were generated as described above, the males were removed, and then the females were maintained in culture vials in the presence or absence of drug for the indicated time period. 5 vials of 20 flies each per treatment were assayed at multiple time points, and total eggs laid over 24 hours per vial were counted using the microscope. The initial data is presented as average eggs per fly per day, and was further quantified using area-under-curve (AUC) analysis in Prism 9 to estimate total eggs laid during the assay period. AUC was conducted for each of the 5 replicate vials, and the average AUC and standard deviation are presented in bar graphs. The difference in AUC between samples was compared using unpaired, two-sided t tests, conducted using Prism 9. Multiple comparisons were controlled using Bonferroni correction, and the *p* value for significance at 5% error rate is indicated in the figure legends.

### PCR analysis of *w[1118]* and *Dhr96[1]* strains

DNA was extracted from three day old *w[1118]* and *Dhr96[1]* strains using the ZymoBIOMICS DNA miniprep kit (Zymo Research # D4300). PCR was performed using primers DHR96-fwd-seq (5’-ATATGGAGCAGCCAGGTGTT-3’) and DHR96-rev (5’-CCACTGGTTCTCAAAGTCAG-3’). The PCR products were resolved by electrophoresis on a 0.8% agarose gel, visualized by ethidium bromide staining, and size measurements of fragments were made by comparison to a 3kbp DNA ladder.

### Maternal *tudor[1]* germ-line ablation

The crossing scheme was carried out as shown (S2 Fig). Briefly, *vas*^*1*^ *cn*^*1*^ *tud*^*1*^ *bw*^*1*^ *speck*^*1*^*/CyO* males were crossed to *tud*^*1*^ *bw*^*1*^ *speck*^*1*^*/CyO*, *l(2)DTS5131* virgins to generate *tud[1]* homozygotes and *tud[1]/CyO* heterozygotes. Virgins of each type were then crossed to *w[1118]* strain males, and *tud[1]/+* heterozygous progeny were collected as virgins from each cross; the *tud[1]* homozygous mothers will generate progeny lacking the germ-line, whereas the *tud[1]/CyO* heterozygous mothers will generate control progeny with normal germ-line. The flies were then assayed as virgin females and mated females, for life span and other phenotypes, as described above.

### Lipidomics analysis

Virgin males, virgin females and mated females of the *w[1118]* strain were generated as described above. The males were removed from the mated groups, and then the virgin males, virgin females and mated females were maintained in culture vials in the presence or absence of 200μg/ml mifepristone (as indicated), from age 2 days to 14 days. Four replicates of 10 flies each for each group were then analyzed by multidimensional mass spectrometry-based shotgun lipidomics for the indicated lipids, and data are expressed after normalization to total protein. Analysis was conducted at the Functional Lipidomics Core, Barshop Institute for Longevity and Aging Studies, University of Texas Health Sciences Center, San Antonio, using published procedures [40–42]. The values are plotted for each of the 4 replicates, with a bar indicating the mean. (-) and (+) drug groups were compared using unpaired, two-sided t test, and *p* values are presented above the plots.

## Supporting information

S1 Fig Backcrossing of *Dhr96[1]* mutation into *w[1118]* background. The *Dhr96[1]* mutation was backcrossed to the *w[1118]* reference strain for 9 generations, by scoring the *3xP3-eGFP* marker, as described in methods. (A-C) A representative female fly from the *w[1118]* reference strain, the backcrossed *Dhr96[1]/+* heterozygotes, and the backcrossed *Dhr96[1]* homozygotes, as indicated. The images are original data and have not been previously published or publicly disclosed. A. Visible light image. B. GFP image. C. Visible light/GFP overlay. D. Map of wild-type *Dhr96* locus. The indicated forward and reverse primers flank exons 4–6, and enable PCR amplification of an expected fragment of 1250 bp. E. Map of *Dhr96[1]* mutant locus. Exon 4 is deleted, and therefore the primers generate an expected fragment of 919 bp. F. Agarose gel electrophoresis and ethidium bromide staining analysis of amplified fragments. MWM, molecular weight markers. NT, no-template control. *w[1118]*, reference strain used for backcrossing. *Dhr96[1]*, homozygous backcrossed *Dhr96[1]* strain. G. Sensitivity to DDT. Virgin females of the *Dhr96[1]* strain and the *w[1118]* strain were assayed for survival in the absence (-) and presence of the indicated concentrations of DDT. For each drug concentration, *Dhr96[1]* is compared to *w[1118]*. Statistical test is log-rank, and the *p* value for significance with one comparison is *p* ≤ 0.05.
(TIFF)

S2 Fig Maternal *tudor[1]* mutation crossing scheme. Long-term maintenance of *tudor[1]* mutation chromosomes over *CyO* balancer results in accumulation of background mutations and a reduction of viable homozygous *tudor[1]* progeny. To overcome this, two different strains bearing the *tudor[1]* mutation were crossed to generate abundant viable *tudor[1]* homozygotes. In step 1, strain BDSC#1735 *vas[1] cn[1] tud[1] bw[1] speck[1]/CyO* males are crossed to strain BDSC#1786 *tud[1] bw[1] speck[1]/CyO* virgins. Non-*Curly* progeny virgins are *tudor[1]* homozygotes, and *Curly* progeny virgins are *tudor[1]/CyO* heterozygotes. In step two, each type of progeny is crossed to *w[1118]* strain males. This generates *tudor[1]* heterozygous virgins that lack the germ-line, and *tudor[1]* heterozygous virgins that contain the germ-line. In step 3, each of these groups is assayed as virgins, and as mated to *w[1118]* males.
(TIFF)

S3 Fig Maternal *tudor[1]* mutation and egg counts from individual vials. (A-F) Each vial contains 20 females. (A, B) Day 8 of drug treatment. (C, D) Day 12 of drug treatment. (E, F) Day 16 of drug treatment. (A, C, E) Maternal *tudor[1]* mutant females, experiment 1. B. Material *tudor[1]* mutant females, experiment 2. VF, virgin female. MF, mated female. (+), 200μg/ml mifepristone. +RH, 1000μg/ml RH5849.
(TIFF)

S4 Fig Egg laying assay. Virgin females of the indicated genotypes were assayed for egg production every other day from days 2 to 34. Data is for 5 replicate vials of 20 flies each, and is plotted as average +/- SD. A. *yw* strain. B. *w[1118]* control strain. C. UAS-GFP strain. D. UAS-TraF strain. E. Progeny of cross *w[1118]* X *yw;ElavGS*. (+), 200μg/ml mifepristone.
(TIFF)

S1 Table *Dhr96[1]* and mifepristone COX-PHA. (DOCX)

S2 Table *Dhr96[1]*, HFD and mifepristone COX-PHA. (DOCX)

S3 Table Lipidomics data. (XLSX)

S4 Table *tudor[1]* and mifepristone COX-PHA. (DOCX)

S5 Table *Mini-white[+]* and mifepristone COX-PHA. (DOCX)

S1 Raw images (PDF)
