# Supplementary figures and images for: Dhr96[1] mutation and maternal tudor[1] mutation increase life span and reduce the beneficial effects of mifepristone in mated female Drosophila
Source: PLoS One. 2023 Dec 21;18(12):e0292820. doi: 10.1371/journal.pone.0292820 (PMC10735022; doi:10.1371/journal.pone.0292820)

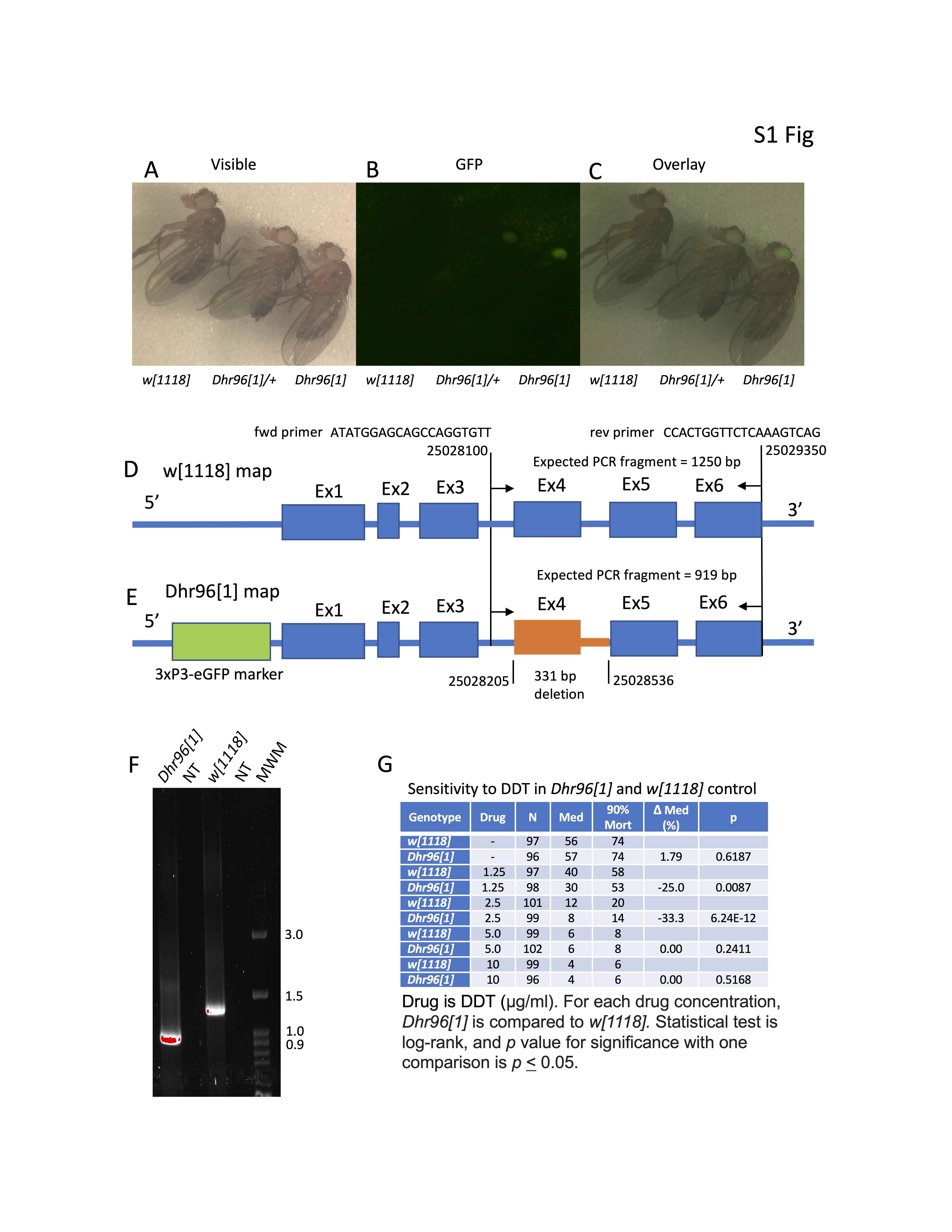

Supplement: S1 Fig — The Dhr96[1] mutation was backcrossed to the w[1118] reference strain for 9 generations, by scoring the 3xP3-eGFP marker, as described in methods. (A-C) A representative female fly from the w[1118] reference strain, the backcrossed Dhr96[1]/+ heterozygotes, and the backcrossed Dhr96[1] homozygotes, as indicated. The images are original data and have not been previously published or publicly disclosed. A. Visible light image. B. GFP image. C. Visible light/GFP overlay. D. Map of wild-type Dhr96 locus. The indicated forward and reverse primers flank exons 4–6, and enable PCR amplification of an expected fragment of 1250 bp. E. Map of Dhr96[1] mutant locus. Exon 4 is deleted, and therefore the primers generate an expected fragment of 919 bp. F. Agarose gel electrophoresis and ethidium bromide staining analysis of amplified fragments. MWM, molecular weight markers. NT, no-template control. w[1118], reference strain used for backcrossing. Dhr96[1], homozygous backcrossed Dhr96[1] strain. G. Sensitivity to DDT. Virgin females of the Dhr96[1] strain and the w[1118] strain were assayed for survival in the absence (-) and presence of the indicated concentrations of DDT. For each drug concentration, Dhr96[1] is compared to w[1118]. Statistical test is log-rank, and the p value for significance with one comparison is p ≤ 0.05. (TIFF) [file pone.0292820.s001.tiff]

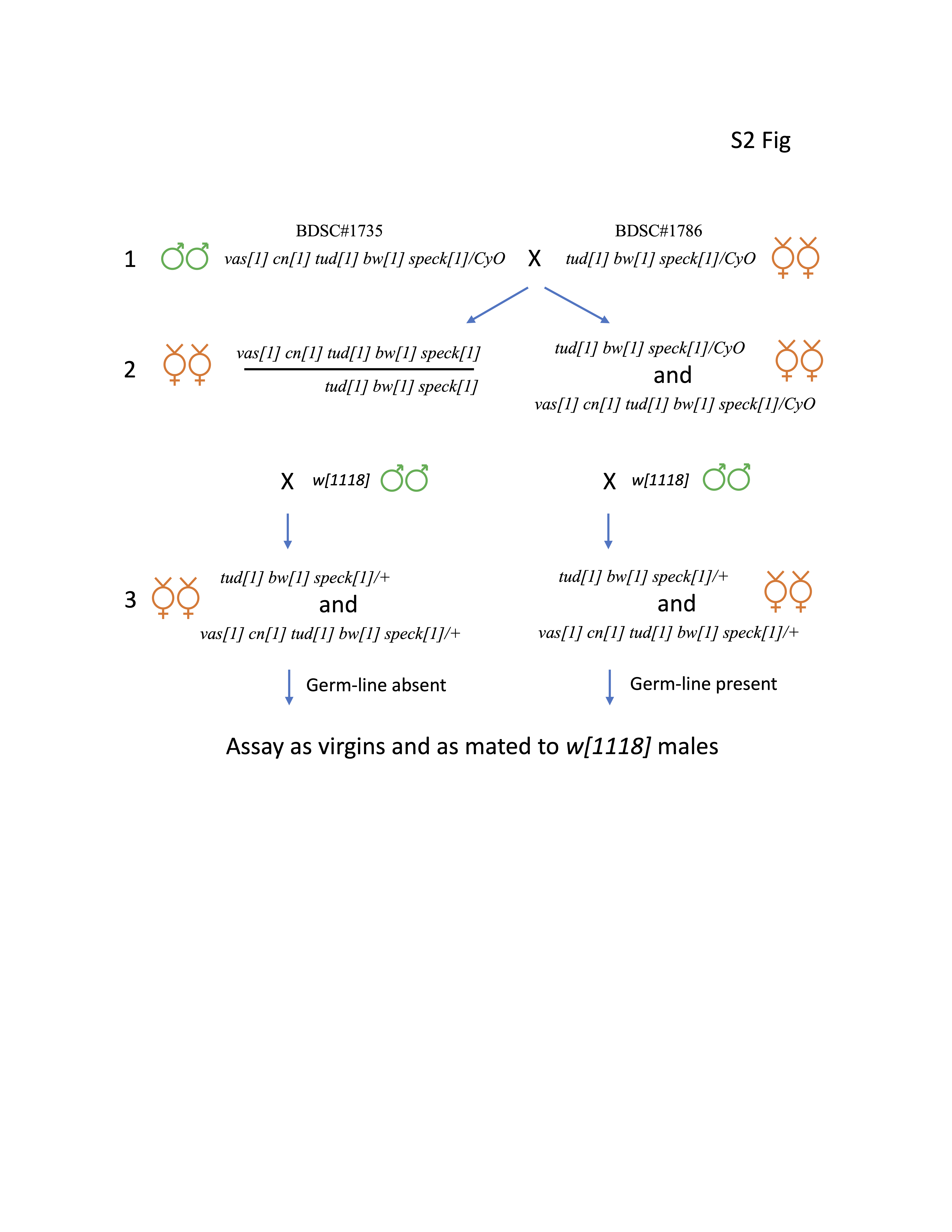

Supplement: S2 Fig — Long-term maintenance of tudor[1] mutation chromosomes over CyO balancer results in accumulation of background mutations and a reduction of viable homozygous tudor[1] progeny. To overcome this, two different strains bearing the tudor[1] mutation were crossed to generate abundant viable tudor[1] homozygotes. In step 1, strain BDSC#1735 vas[1] cn[1] tud[1] bw[1] speck[1]/CyO males are crossed to strain BDSC#1786 tud[1] bw[1] speck[1]/CyO virgins. Non-Curly progeny virgins are tudor[1] homozygotes, and Curly progeny virgins are tudor[1]/CyO heterozygotes. In step two, each type of progeny is crossed to w[1118] strain males. This generates tudor[1] heterozygous virgins that lack the germ-line, and tudor[1] heterozygous virgins that contain the germ-line. In step 3, each of these groups is assayed as virgins, and as mated to w[1118] males. (TIFF) [file pone.0292820.s002.tiff]

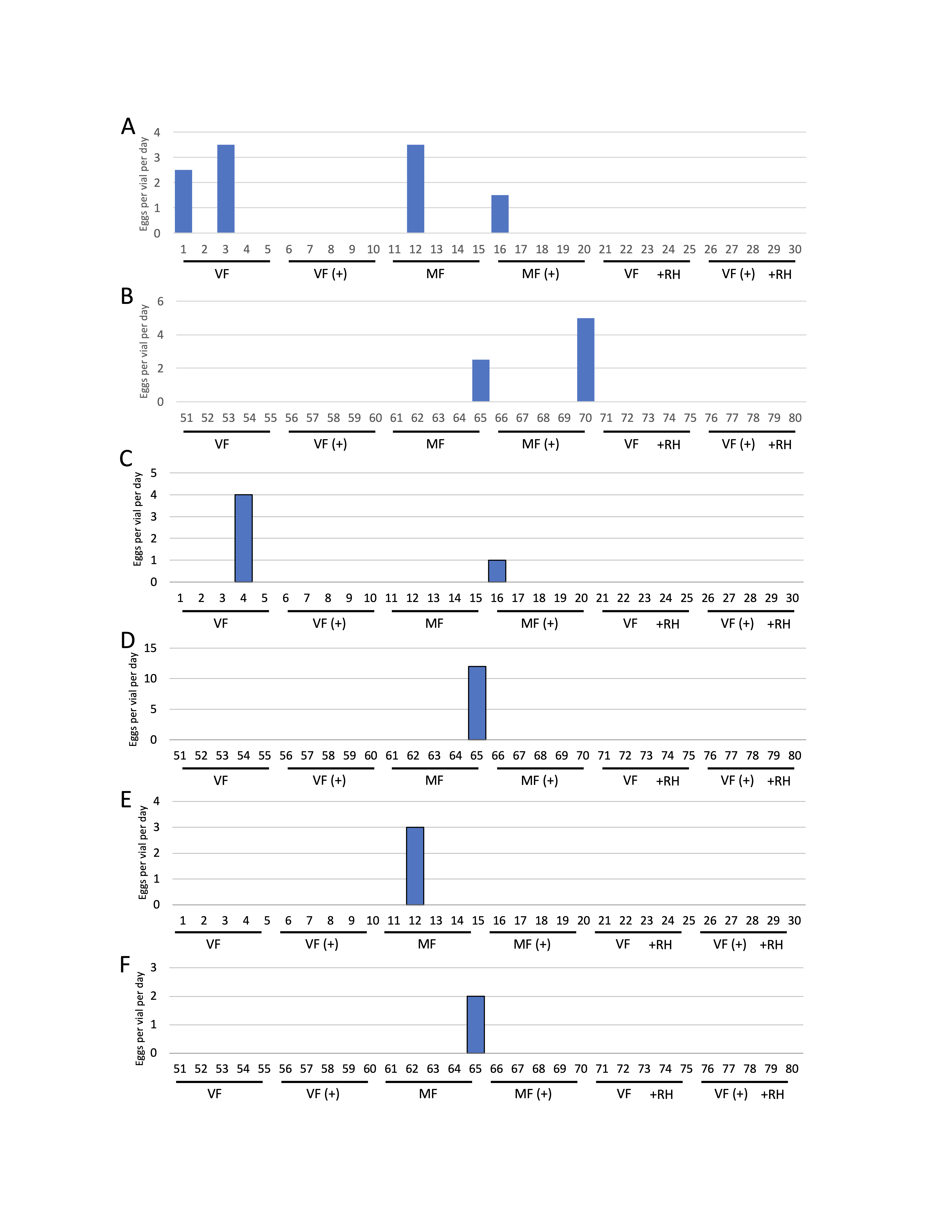

Supplement: S3 Fig — (A-F) Each vial contains 20 females. (A, B) Day 8 of drug treatment. (C, D) Day 12 of drug treatment. (E, F) Day 16 of drug treatment. (A, C, E) Maternal tudor[1] mutant females, experiment 1. B. Material tudor[1] mutant females, experiment 2. VF, virgin female. MF, mated female. (+), 200μg/ml mifepristone. +RH, 1000μg/ml RH5849. (TIFF) [file pone.0292820.s003.tiff]

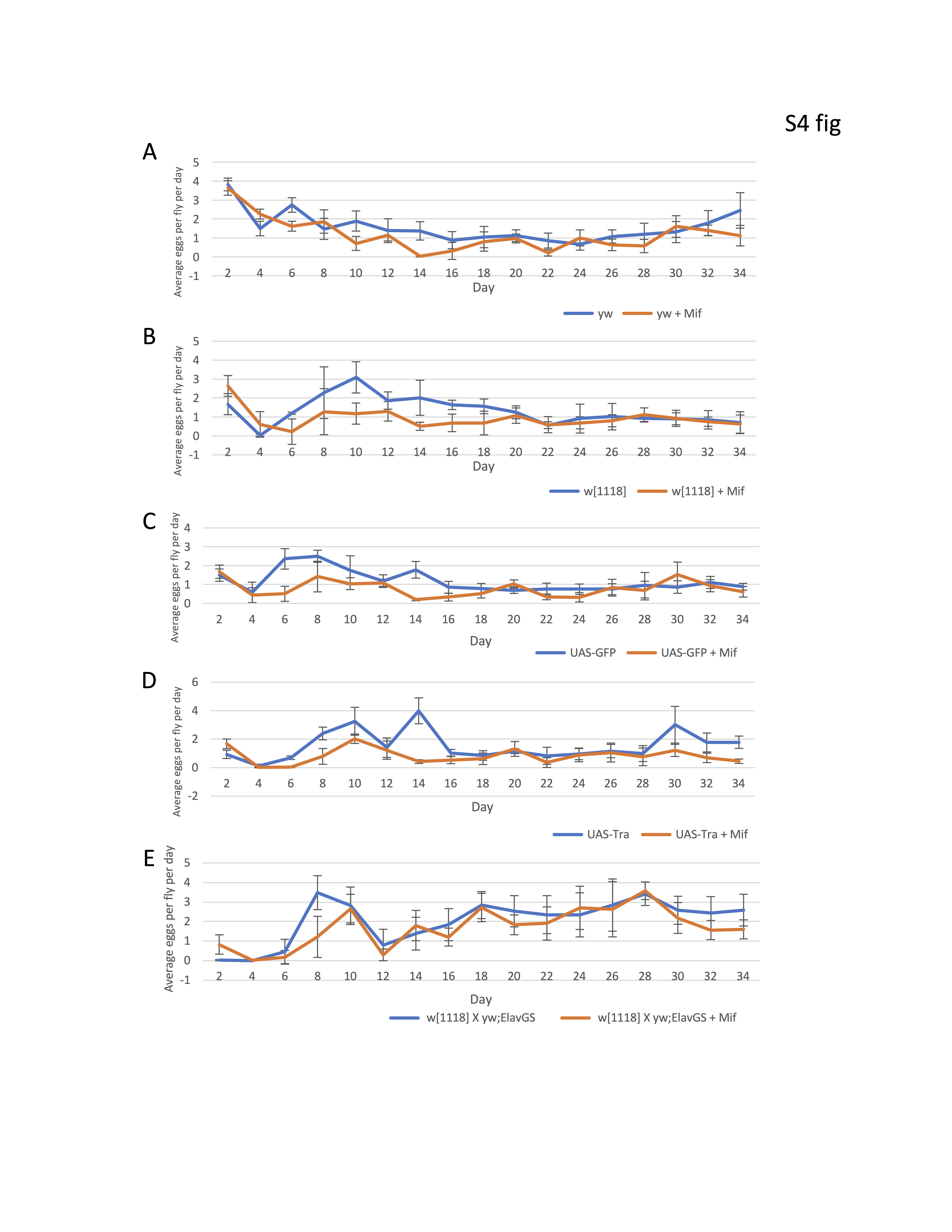

Supplement: S4 Fig — Virgin females of the indicated genotypes were assayed for egg production every other day from days 2 to 34. Data is for 5 replicate vials of 20 flies each, and is plotted as average +/- SD. A. yw strain. B. w[1118] control strain. C. UAS-GFP strain. D. UAS-TraF strain. E. Progeny of cross w[1118] X yw;ElavGS. (+), 200μg/ml mifepristone. (TIFF) [file pone.0292820.s004.tiff]
