# Supplementary material for: Dhr96[1] mutation and maternal tudor[1] mutation increase life span and reduce the beneficial effects of mifepristone in mated female Drosophila
Source: PLoS One. 2023 Dec 21;18(12):e0292820. doi: 10.1371/journal.pone.0292820 (PMC10735022; doi:10.1371/journal.pone.0292820)
Supplement: S1 Table — (DOCX) [file pone.0292820.s005.docx]

S1 Table. *Dhr96[1]* and mifepristone COX-PA

Call: coxph(formula = (Surv(Day) ~ Mif + Mating + DHR96 + Mif:Mating +

Mif:DHR96 + DHR96:Mating), data = dataset)

n= 3102, number of events= 3102

coef exp(coef) se(coef) z Pr(>|z|)

Mif -0.06988 0.93250 0.06206 -1.126 0.26016

Mating 0.37064 1.44866 0.06261 5.920 3.22e-09 ***

DHR96 -0.51284 0.59879 0.06324 -8.110 5.08e-16 ***

Mif:Mating -0.51566 0.59711 0.07279 -7.084 1.40e-12 ***

Mif:DHR96 0.19130 1.21083 0.07237 2.643 0.00821 **

Mating:DHR96 0.30078 1.35092 0.07210 4.172 3.03e-05 ***

---

Signif. codes: 0 ‘***’ 0.001 ‘**’ 0.01 ‘*’ 0.05 ‘.’ 0.1 ‘ ’ 1

exp(coef) exp(-coef) lower .95 upper .95

Mif 0.9325 1.0724 0.8257 1.0531

Mating 1.4487 0.6903 1.2814 1.6378

DHR96 0.5988 1.6700 0.5290 0.6778

Mif:Mating 0.5971 1.6747 0.5177 0.6887

Mif:DHR96 1.2108 0.8259 1.0507 1.3954

Mating:DHR96 1.3509 0.7402 1.1729 1.5560

Concordance = 0.555 (se = 0.006 )

Likelihood ratio test = 199.3 on 6 df, p=<2e-16

Wald test = 202.8 on 6 df, p=<2e-16

Score (logrank) test = 207.6 on 6 df, p=<2e-16
