# Supplementary material for: Dhr96[1] mutation and maternal tudor[1] mutation increase life span and reduce the beneficial effects of mifepristone in mated female Drosophila
Source: PLoS One. 2023 Dec 21;18(12):e0292820. doi: 10.1371/journal.pone.0292820 (PMC10735022; doi:10.1371/journal.pone.0292820)
Supplement: S2 Table — (DOCX) [file pone.0292820.s006.docx]

S2 Table. *Dhr96[1]*, HFD and mifepristone COX-PA

Call: coxph(formula = (Surv(Day) ~ Mif + DHR96 + CO + Mif:DHR96 + DHR96:CO +

Mif:CO), data = newfile_combined)

n= 1188, number of events= 1188

coef exp(coef) se(coef) z Pr(>|z|)

Mif -0.17204 0.84194 0.11075 -1.553 0.12031

DHR96 -0.09715 0.90742 0.11063 -0.878 0.37988

CO 0.58986 1.80373 0.02885 20.443 < 2e-16 ***

Mif:DHR96 0.09728 1.10217 0.11695 0.832 0.40554

DHR96:CO 0.08731 1.09124 0.02908 3.002 0.00268 **

Mif:CO 0.02343 1.02371 0.02851 0.822 0.41108

---

Signif. codes: 0 ‘***’ 0.001 ‘**’ 0.01 ‘*’ 0.05 ‘.’ 0.1 ‘ ’ 1

exp(coef) exp(-coef) lower .95 upper .95

Mif 0.8419 1.1877 0.6777 1.046

DHR96 0.9074 1.1020 0.7305 1.127

CO 1.8037 0.5544 1.7046 1.909

Mif:DHR96 1.1022 0.9073 0.8764 1.386

DHR96:CO 1.0912 0.9164 1.0308 1.155

Mif:CO 1.0237 0.9768 0.9681 1.083

Concordance = 0.769 (se = 0.007 )

Likelihood ratio test = 1009 on 6 df, p=<2e-16

Wald test = 884.4 on 6 df, p=<2e-16

Score (logrank) test = 1052 on 6 df, p=<2e-16
