# Supplementary material for: Dhr96[1] mutation and maternal tudor[1] mutation increase life span and reduce the beneficial effects of mifepristone in mated female Drosophila
Source: PLoS One. 2023 Dec 21;18(12):e0292820. doi: 10.1371/journal.pone.0292820 (PMC10735022; doi:10.1371/journal.pone.0292820)
Supplement: S4 Table — (DOCX) [file pone.0292820.s008.docx]

SX Table. *tudor[1]* and mifepristone COX-PA

Call: coxph(formula = (Surv(Day) ~ Mif + Mating + Tudor + Mif:Mating +

Mif:Tudor + Tudor:Mating), data = Tudor_dataset)

n= 1512, number of events= 1512

coef exp(coef) se(coef) z Pr(>|z|)

Mif -1.05836 0.34702 0.09685 -10.928 < 2e-16 ***

Mating 1.02381 2.78379 0.09061 11.299 < 2e-16 ***

Tudor 1.09959 3.00292 0.09595 11.460 < 2e-16 ***

Mif:Mating -0.44091 0.64345 0.10419 -4.232 2.32e-05 ***

Mif:Tudor 0.70629 2.02646 0.10995 6.424 1.33e-10 ***

Mating:Tudor -0.58604 0.55653 0.10443 -5.612 2.00e-08 ***

---

Signif. codes: 0 ‘***’ 0.001 ‘**’ 0.01 ‘*’ 0.05 ‘.’ 0.1 ‘ ’ 1

Exp(coef) exp(-coef) lower .95 upper .95

Mif 0.3470 2.8817 0.2870 0.4196

Mating 2.7838 0.3592 2.3308 3.3248

Tudor 3.0029 0.3330 2.4881 3.6242

Mif:Mating 0.6435 1.5541 0.5246 0.7892

Mif:Tudor 2.0265 0.4935 1.6336 2.5138

Mating:Tudor 0.5565 1.7969 0.4535 0.6829

Concordance = 0.666 (se = 0.009 )

Likelihood ratio test = 658.8 on 6 df, p=<2e-16

Wald test = 562 on 6 df, p=<2e-16

Score (logrank) test = 634.4 on 6 df, p=<2e-16
