# Supplementary material for: Dhr96[1] mutation and maternal tudor[1] mutation increase life span and reduce the beneficial effects of mifepristone in mated female Drosophila
Source: PLoS One. 2023 Dec 21;18(12):e0292820. doi: 10.1371/journal.pone.0292820 (PMC10735022; doi:10.1371/journal.pone.0292820)
Supplement: S5 Table — (DOCX) [file pone.0292820.s009.docx]

SX Table. *Mini-white[+]* and mifepristone COX-PA

Call: coxph(formula = (Surv(Day) ~ Mif * Miniwhite), data = newfile_combined)

n= 1185, number of events= 1185

coef exp(coef) se(coef) z Pr(>|z|)

Mif -0.15034 0.86042 0.10036 -1.498 0.134

Miniwhite -0.52836 0.58957 0.08872 -5.955 2.6e-09 ***

Mif:Miniwhite 0.06078 1.06267 0.12334 0.493 0.622

---

Signif. codes: 0 ‘***’ 0.001 ‘**’ 0.01 ‘*’ 0.05 ‘.’ 0.1 ‘ ’ 1

exp(coef) exp(-coef) lower .95 upper .95

Mif 0.8604 1.162 0.7068 1.0475

Miniwhite 0.5896 1.696 0.4955 0.7015

mif:Miniwhite 1.0627 0.941 0.8345 1.3533

Concordance = 0.567 (se = 0.009 )

Likelihood ratio test = 62.47 on 3 df, p=2e-13

Wald test = 66.13 on 3 df, p=3e-14

Score (logrank) test = 67.57 on 3 df, p=1e-14
