# Supplementary material for: Dhr96[1] mutation and maternal tudor[1] mutation increase life span and reduce the beneficial effects of mifepristone in mated female Drosophila
Source: PLoS One. 2023 Dec 21;18(12):e0292820. doi: 10.1371/journal.pone.0292820 (PMC10735022; doi:10.1371/journal.pone.0292820)

Original image used to generate S1 Fig panel F
The original image is a TIFF generated by the Bio Rad Geldoc imaging system.
The image is of an agarose electrophoresis gel stained with ethidium bromide.
X indicates lanes that were not used and were cropped to generate the final figure.

Dhr96[1] is the Dhr96[1] mutant strain DNA
NT is no-template control PCR reaction
w[1118] is the isogenic control strain DNA
MWM is molecular weight markers

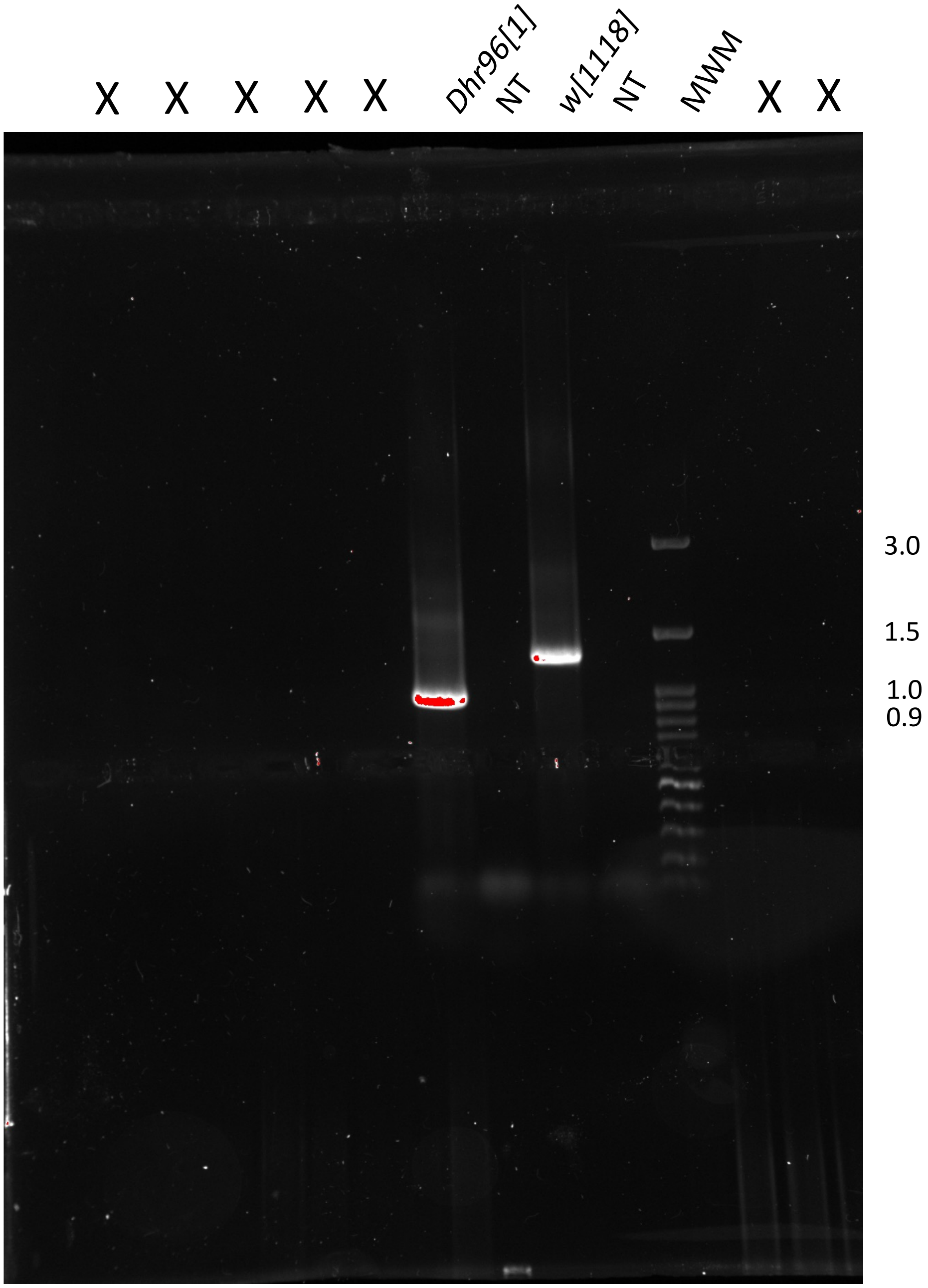

Supplement: S1 Raw images — (PDF) [file pone.0292820.s010.pdf]
